# Association between COVID-19 infection and new-onset dementia in older adults: a systematic review and meta-analysis

**DOI:** 10.1186/s12877-024-05538-5

**Published:** 2024-12-15

**Authors:** Dan Shan, Congxiyu Wang, Trevor Crawford, Carol Holland

**Affiliations:** 1 Centre for Ageing Research, Division of Health Research, Faculty of Health and Medicine, Lancaster University, Health Innovation Campus, Sir John FisBailrigg, Lancasterher Drive, Bailrigg, Lancaster, LA1 4YT UK; 2 Department of Psychiatry, University of Oxford, Oxford, UK; 3 Centre for Ageing Research, Department of Psychology, Faculty of Science and Technology, Lancaster University, Lancaster, UK

**Keywords:** Alzheimer’s disease, COVID-19, Dementia, Meta‐analysis, Older adults, Review, Respiratory infection

## Abstract

**Background:**

The relationship between COVID-19 infection and a possible increased likelihood of older adults developing new-onset dementia (NOD) remains elusive.

**Methods:**

A thorough search was performed across several databases including MEDLINE/PubMed, PsycINFO, Scopus, medRxiv, and PQDT Global for studies published in English from January 2020 to December 2023. Only original investigations exploring the link between COVID-19 infection and NOD were selected for inclusion. We assessed the risk of developing NOD, using Risk Ratio (RR) for measurement. Control groups were categorized as: (i) a non-COVID cohort with other respiratory infections [control group (C1)]; and (ii) a non-COVID cohort with otherwise unspecified health status [control group (C2)]. Follow-up periods were divided into intervals of 3, 6, 12, and 24 months post-COVID.

**Results:**

11 studies (involving 939,824 post-COVID-19 survivors and 6,765,117 controls) were included in the review. Across a median observation period of 12 months post-COVID, the overall incidence of NOD was about 1.82% in the COVID-infected group, compared to 0.35% in the non-COVID-infected group. The overall pooled meta-analysis showed a significantly increased NOD risk among COVID-19 older adult survivors compared to non-COVID-19 controls (RR = 1.58, 95% CI 1.21–2.08). Similar increased NOD risks were observed in subgroup analyses restricted to an observational period of 12 months (RR = 1.56, 95% CI 1.21–2.01), as well as in five studies that employed propensity score matching to sufficiently and effectively control for multiple confounding covariates (RR = 1.46, 95% CI 1.10–1.94). COVID-19 group and C1 group shared a comparably increased risk of developing NOD (overall RR = 1.13, 95% CI 0.92–1.38).

**Discussion:**

Under normal circumstances, we believe that COVID-19 infection is likely to be a risk factor for developing NOD in older adults over time. While the increased NOD risk due to COVID-19 infection appears to be similar to that associated with other respiratory infections, it warrants and necessitates investigation with longer observations.

**Supplementary Information:**

The online version contains supplementary material available at 10.1186/s12877-024-05538-5.

## Introduction

The COVID-19 pandemic, precipitated by the emergence of the novel coronavirus SARS-CoV-2, has profoundly disrupted global health paradigms, extending its influence beyond acute illness to potentially shape long-term neurological trajectories [1]. Among the most scrutinized consequences in recent years is the heightened risk of cognitive impairment and the emergence or exacerbation of neurodegenerative conditions [2, 3], including Alzheimer’s disease and other types of dementia [4, 5], in older adults following COVID-19 infection [6].

Emerging research has increasingly drawn attention to the correlation between COVID-19 infection and escalated risks of cognitive decline or “brain fog” in older adults, in comparison with those affected by other respiratory diseases, or with healthy older adults who are otherwise characteristic-matched [6–11]. Some evidence suggests that COVID-19 may precipitate the new-onset of Alzheimer’s disease and other dementias, or exacerbate pre-existing neurodegenerative conditions [12, 13]. This conjecture is bolstered by prior neurobiological studies illustrating how SARS-CoV-2 could trigger central nervous system inflammation and dysregulation, trigger autoimmune responses detrimental to neurological function, and potentially expedite neurodegenerative processes [14]. For instance, COVID-19 has been linked to the activation of the NLRP3 inflammasome, tau aggregation, neurodegeneration, and elevated levels of amyloid-beta deposition and cerebrospinal fluid markers such as neurofilament light chain, and tau, suggesting ties to Alzheimer’s disease pathology [15]. Also, COVID-19’s role in cerebral ischemia, thrombus formation, and hypoxia aligns with vascular dementia mechanisms [16]. Additionally, in populations with an elevated baseline dementia risk, particularly older adults with cardiovascular risk factors, COVID-19 not only augments cognitive decline risks but also synergistically interacts with pre-existing dementia risk factors, leading to a disproportionate escalation in dementia risk [16].

Despite these explorations, the literature examining the link between COVID-19 and dementia new-onset remains fragmented, characterised by diverse methodologies and nuanced outcomes. These variabilities span research approaches, baseline clinical characteristics of COVID-19-afflicted patients, comparator groups, follow-up durations, dementia types, ethnic demographics and so on. While a previous meta-analysis by Rahmati et al. examined this link across participants of all ages [17], no clear association among older adults has been established. Rahmati et al. simplistically pointed out that individuals aged 65 and older who contracted COVID-19 faced a higher risk of NOD compared to those without infection under 65. However, detailed comparisons within subgroups—such as by sex, type of respiratory infection, severity of COVID-19, pre-existing medical conditions, and duration of follow-up among the older adults—remain unclear from existing studies. Considering the differences in cognitive reserve between young and older adults (e.g., aging being a significant risk factor for cognitive decline and dementia), it is crucial to distinguish between these age groups in analyses. Consequently, there is a notable void in the systematically exploration and in quantifying COVID-19’s association with Alzheimer’s disease and other dementias in older adults over time following acute COVID-19 infection. This understanding is crucial, as evidence has suggested the devastating impacts of dementia, affecting not only the patients themselves, leading to a grim prognosis (e.g., elevated mortality risks) [18, 19], but also their families, significantly increasing social, medical, and economic burdens [20]. Furthermore, the ongoing pandemic and associated potential for multiple infections in vulnerable older adults, as well as the projected tripling of the global dementia burden by 2050 [21], underscore the urgency of this inquiry.

This review endeavoured to bridge these research gaps by rigorously analysing extant original investigation studies, thereby offering a more definitive comprehension of these associations. This effort was geared towards enhancing the management and care of COVID-19-infected older adults as well as their long-term cognitive care and, fostering early intervention strategies in the waning, yet still unpredictable pandemic era. The primary objective was to ascertain the degree to which the COVID-19 infection could impact the risk of subsequent NOD development over time in older adults. When possible, secondary objectives included investigating the impacts of demographic and health-related factors (e.g., the type of respiratory infection, sex difference, and the severity of COVID-19) on the NOD risk. We also aimed to examine cognitive impairment, including cases of cognitive impairment no dementia (CIND) and dementia, as an outcome induced by COVID-19 infection.

## Methods & materials

In this systematic review and meta-analysis, we adhered to the Preferred Reporting Items for Systematic Reviews and Meta-Analyses (PRISMA) 2020 guideline. Its checklist is detailed in Supplementary File 1 (Table S1) [22]. Furthermore, this review was registered with the International Prospective Register of Systematic Reviews (PROSPERO; reference number CRD42023491714) prior to its commencement.

### Search strategy and selection criteria

We conducted comprehensive electronic searches across five major databases: MEDLINE/PubMed, APA PsycINFO, Scopus, medRxiv preprint server, and ProQuest Dissertations and Theses Global (PQDT Global), targeting English-language publications from January 2020 to December 2023. Our systematic review and meta-analysis adhered to predefined inclusion criteria focused on participants’ characteristics (P), Exposure (E), and outcomes (O). Participants (P) were older adults aged 60 and above, with or without COVID-19 infection. The exposure (E), in the observational context, was COVID-19 infection. The main outcome (O) was the incidence of new-onset dementia (NOD): in individuals with COVID-19 infection, NOD was diagnosed after the infection; in individuals without COVID-19 infection, NOD was diagnosed after the beginning of the study observation period. This distinction allows us to evaluate the impact of COVID-19 infection on NOD risk compared to those without infection.

A list of keywords associated with COVID-19 and its neurological impacts was used, simplifying the search algorithm to (“COVID-19” OR “SARS-CoV-2” OR “coronavirus” OR “pandemic” OR “post-COVID syndrome” OR “long COVID” OR “chronic COVID”) AND (“Alzheimer’s Disease” OR “dementia” OR “neurodegenerative disorder” OR “neurodegeneration” OR “neurological sequelae” OR “brain health”) AND (“older adult” OR “elderly” OR “geriatric” OR “senior population” OR “aging” OR “ageing”). For comprehensive details of our search algorithm in the searched databases, see Table S2.

Regarding the inclusion criteria for our study, eligible article types included original empirical articles, short communications, and research letters. Our selection was limited to studies evaluating the impact of COVID-19 infection on the new-onset of any type of dementia among survivors aged 60 years and older (i.e., older adults) [23], with longitudinal observations. New-onset dementia in our study refers to cases where dementia was diagnosed after COVID-19 infection in the COVID-19-infected cohort, and after the official start of the original studies in the non-COVID-19-infected group, with both groups having no prior history of dementia. Any study failing to meet these criteria were excluded. Additionally, original studies that did not clearly differentiate between COVID-19 and non-COVID-19 groups within the same research were excluded. Studies evaluating all age groups were also excluded if data on older adults could not be separated. We manually screened some retrieved review studies to identify additional eligible empirical research not captured in our initial search. We also conducted manual searches of reference lists from both the articles and reviews obtained to find more relevant studies.

Overall, we considered observational studies involving older adults (≥ 60) who had recovered from COVID-19 (forming the exposure group) and underwent dementia assessments at certain stages post-recovery. In our evaluation of comparison groups within potentially eligible studies, all non-COVID status age-matched individuals were considered as controls, including both healthy participants and participants with other types of respiratory infections. EndNote 21 software (Clarivate Analytics) was used for literature management.

### Data extraction, planned subgroup analysis, and research quality assessment

The following data were extracted from the eligible studies: author(s) and publication year, study country, research type, description of exposure and control groups (i.e., demographic and health-related characteristics of both COVID-19 survivors and controls, where available), participants’ age range, COVID-19 diagnosis methodology and setting, dementia determination method and setting, type of dementia examined, and observational period. The counts of events (e.g., diagnosed dementia) and non-events in both the COVID and non-COVID older adult groups were either directly recorded or calculated from the data presented in the original articles or their accompanying supplementary files. When a study investigated multiple follow-up periods post-COVID-19, the longest follow-up duration was retained for our main analysis (i.e., the overall pooled meta-analysis).

For performing subgroup analyses, when accessible, the collected information about demographic characteristics included age group (such as 60–69, 70–79, 80–89, ≥ 90) and sex (male, female). Health-related characteristics included the type of newly developed dementia (all-cause dementia, Alzheimer’s Disease, vascular dementia, Lewy Body dementia, and others), type of respiratory infection (COVID-19, influenza A/B, and bacterial infection), co-morbidities, COVID-19 severity status (inpatient vs. outpatient), indication of statistically significant cognitive impairment (including both CIND and dementia cases), and follow-up duration (3, 6, 12, 24 months). Here we subjectively considered COVID-19 older adult patients requiring hospitalization as severe cases, while outpatient cases were considered non-severe.

To guarantee the reliability and validity of our findings, we assessed the quality of the included studies by evaluating their risk of bias using the nine-star Newcastle–Ottawa Scale (NOS). This scale is recognized for its effectiveness in appraising the quality of non-randomized studies (e.g., observational studies) in systematic reviews with meta-analysis. Studies scoring more than seven on the NOS were considered high quality, signifying their appropriateness for inclusion in our review [24].

Two assessors (D.S. and C.X.Y.W.) independently conducted a quality appraisal of the included articles. Any potential disagreements would be resolved through discussions with two additional reviewers (C.H. and T.C.) before proceeding to the meta-analysis.

### Statistical analyses

Binary outcome comparisons between a COVID-19 infected group and a non-COVID-19 control group (which included individuals with other respiratory infections or otherwise unspecified health status) were pooled and analysed, with results presented as risk ratios (RR) and 95% confidence intervals (CI). The binary outcome measured was the presence or absence of new-onset dementia at follow-up periods of 3, 6, 12, and 24 months post-infection. The reported log RRs were converted back to RRs through exponentiation. To estimate pooled effect sizes, random-effects models employing the Restricted Maximum Likelihood (REML) method were used for more accurate variance component estimation across studies, therefore enhancing the generalizability of the model’s findings [25]. The potential presence of heterogeneity beyond sampling error was examined using Cochran’s Q statistics and I^2^ statistics. The I^2^ values were categorized as low (< 25%), low to moderate (25–50%), moderate to substantial (50–75%), or substantial (> 75%) [25]. Visual analysis of between-study variance was supported by L’Abbé and Galbraith plots. A random-effects meta-regression model was utilized to identify variables potentially causing significant between-study variance. The robustness of summary estimates and the influence of individual studies on heterogeneity were assessed using a leave-one-out sensitivity analysis [26]. Publication bias was evaluated through a contour-enhanced funnel plot, combined with Egger’s regression test, and Begg’s rank correlation test (for the number of included studies exceeding ten). We used the Trim-and-fill method to provide the adjusted effect sizes including imputed studies. All meta-analyses were conducted in Stata 18.0, considering a two-tailed p value of less than 0.05 as statistically significant.

## Results

### Study screening and general characteristics of include studies

**Fig. 1:**
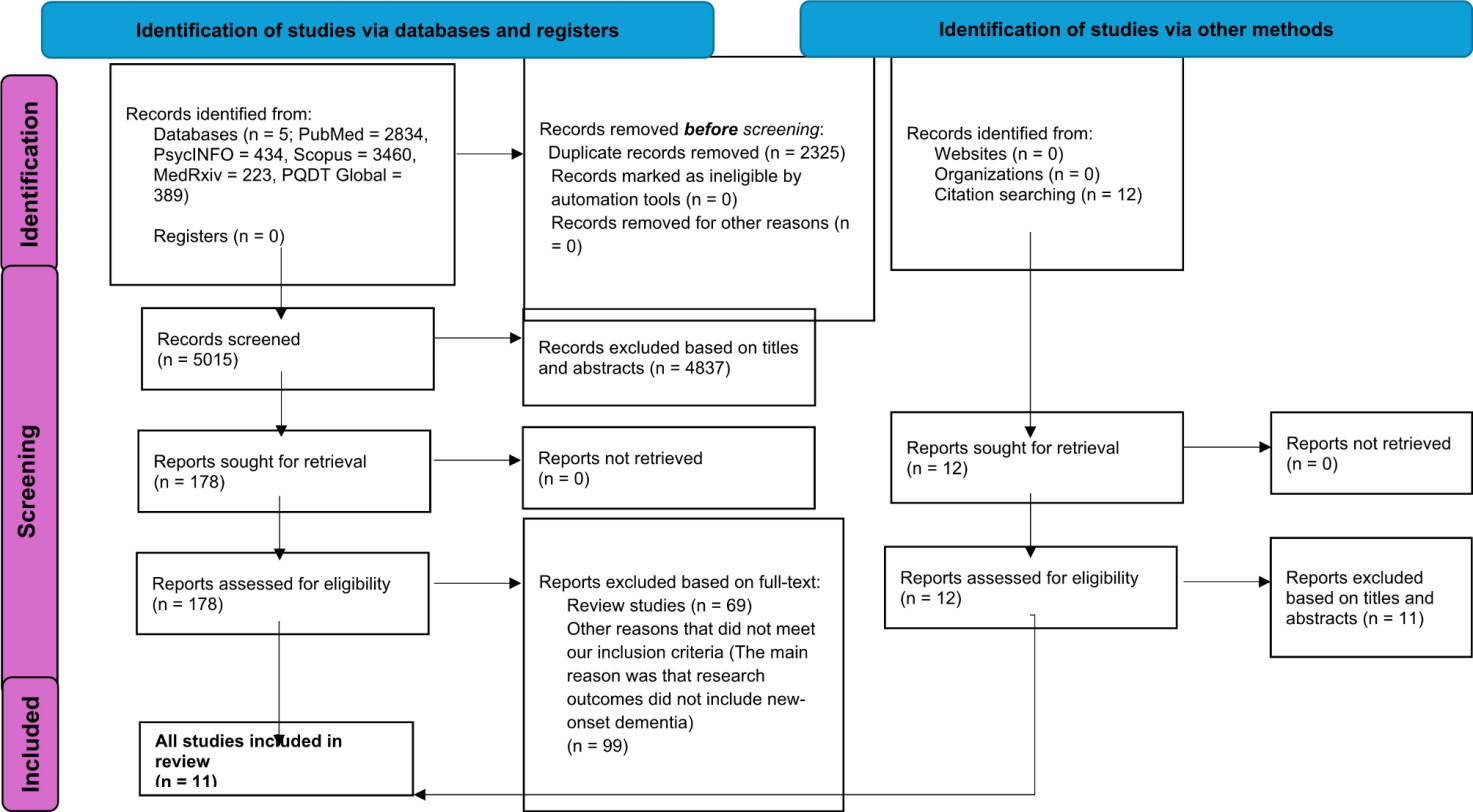
Preferred Reporting Items for Systematic Reviews and Meta-Analyses (PRISMA) diagram demonstrating search strategy

Our systematic review and meta-analysis initiated with the process of literature search and screening. The PRISMA flow diagram of the included studies is presented in Fig. 1.

During the literature screening process, D.S. took the lead role in making inclusion decisions, both at the ‘title and abstract stage’ and the ‘full text screening stage,’ in consultation with other researchers. No significant disagreement between authors was found in this process. Table S3 lists the studies that investigated the associations of our interests from certain perspectives yet were excluded due to their deviation from our review’s precise scope, including the reasons for their exclusion.

This procedure consequently resulted in the inclusion of data from 939,824 post-COVID-19 cases and 6,765,117 controls across 11 studies [6, 9, 12, 13, 27–33], as detailed in Table 1. All these studies investigated the risk of NOD in older adults with and without COVID-19 infection over varying observational periods. The overall incidence of NOD in the COVID-infected group was around 1.82% (ranging from 0.3 to 6.4%), while the non-COVID-infected group exhibited an incidence of around 0.35% (ranging from 0.0 to 5.0%), over a median observation period of 12 months (ranging from 3 to 24 months). To interpret, the overall incidence was calculated by aggregating the number of NOD cases and the total number of individuals in the COVID-19-infected or non-COVID-infected groups across all included studies to obtain an averaged incidence. For example, to calculate the averaged overall incidence in the COVID-infected group, we summed the NOD cases specifically from the COVID-infected group from each study and divided them by the total number of participants in the COVID-infected groups. We applied the same method to calculate the averaged overall incidence in the non-COVID-infected group, and the incidence in individual studies. In studies reporting incidences for different follow-up periods (e.g., 3, 6, 12, or 24 months), we used the longest available follow-up period for consistency.

**Table 1:** A summary of the general characteristics of the 11 studies included in this review

Study	Country	Type of research	Groups	Older adults age group	COVID-19 diagnosis	COVID-19 diagnosis setting	Dementia assessment	Dementia diagnosis setting	Examined type of dementia	Post-COVID follow-up
Cohen et al. (2022)	USA	Retrospective cohort	COVID +: 87,337 Matched COVID -: 87,337	≥ 65 years	RT-PCR	Inpatient and outpatient	ICD-10	Hospital	All-cause Dementia	6 months
Gollop et al. (2023)	Germany	Retrospective cohort	COVID+: 8129 Matched AURI: 8129	≥ 65 years	RT-PCR	General practitioner (GP)	ICD-10	General practitioner (GP)	All-cause Dementia	3, 6, and 12 months
Liu et al. (2021)	China Mainland	*Cross-sectional	COVID +: 1539 COVID -: 466	≥ 60 years	RT-PCR	Inpatient and outpatient	TICS-40	Remote	All-cause Dementia	6 months
Liu et al. (2022)	China Mainland	Prospective cohort	COVID +: 1438 COVID -: 438	≥ 60 years	RT-PCR	Inpatient and outpatient	TICS-40	Remote	All-cause Dementia	12 months
Park et al. (2021)	South Korea	Retrospective cohort	COVID +: 1694 COVID -: 82,164	≥ 60 years	RT-PCR	Inpatient and outpatient	ICD-10	Hospital	All-cause Dementia	6 months
Qureshi et al. (2022)	USA	Retrospective cohort	COVID + pneumonia: 3558 Matched COVID - pneumonia: 3538	> 70 years	RT-PCR	Inpatient and outpatient	ICD-10	Hospital	All-cause Dementia	12 months
Taquet et al. (2021)	USA	Retrospective cohort	COVID +: 15,806 Matched Influenza: 4416 Matched other respiratory tract infection: 13,326	≥ 65 years	RT-PCR	Inpatient and outpatient	ICD-10	Primary care setting and Hospital	All-cause Dementia	3 months
Taquet et al. (2022)	USA, Australia, the UK, Spain, Bulgaria, India, Malaysia, and Taiwan	Retrospective cohort	COVID+: 242,101 Matched another respiratory infection: 242,101	≥ 65 years	RT-PCR	Inpatient and outpatient	ICD-10	Primary care setting and Hospital	All-cause Dementia	6 and 24 months
Wang et al. (2022)	USA	Retrospective cohort	COVID+: 410,478 Matched COVID-: 410,478	≥ 65 years	RT-PCR	Inpatient and outpatient	ICD-10	Primary care setting and Hospital	AD	12 months
Xu et al. (2022)	USA	Retrospective cohort	COVID+: 154,068 Contemporary COVID-: 5,638,795	≥ 60 years	RT-PCR	Inpatient and outpatient	ICD-10	Primary care setting and Hospital	AD	12 months
Zarifkar et al. (2022)	Denmark	Retrospective cohort	COVID+: 13,676 COVID-: 270,023 Influenza A/B: 3906	≥ 60 years	RT-PCR	Inpatient and outpatient	ICD-10	Primary care setting and Hospital	AD	3, 6 and 12 months

*Longitudinal in nature with a cross-sectional analysis

Of note, five studies employed Propensity Score Matching (PSM) to establish 1:1 matched cohorts of older adults without COVID-19, therefore ensuring comparability of baseline characteristics between COVID-positive and control groups [12, 27, 28, 30, 32]. The dementia risk in the COVID-positive group was compared to two types of control groups: non-COVID cohorts with other respiratory infections [control group (C1)] [12, 28, 30, 31, 33], and non-COVID cohorts with otherwise unspecified health status [control group (C2)] [6, 9, 13, 27, 29, 32, 33]. In addition, while nine studies recorded definitive dementia diagnoses using ICD-10, the TICS-40 in Liu et al.‘s studies was used to indicate, rather than confirm, dementia [6, 9]. The focus was on all-cause dementia (primarily including AD, vascular dementia, and unspecified dementia) in eight studies, while the remaining three specifically examined AD [13, 32, 33]. In studies addressing all-cause dementia, AD was the most prevalent type (if they reported the proportions of each dementia subtype), followed by vascular dementia [29, 31]. Refer to Table 1 for more details of other characteristics.

### Quality assessment

Every study included in our review was rated as good quality (≥ 7 stars) based on the NOS quality assessment criteria [34], as the details shown in Table S4(a) for 10 cohort studies and Table S4(b) for one cross-sectional study (longitudinal in nature with a cross-sectional analysis). No disagreement regarding the quality appraisal among included studies between researchers was found. The studies by Park et al. and Qureshi et al. each lost one point for NOS comparability items, as they only controlled for demographic features, but not for known dementia risk factors such as the body mass index, alcohol consumption, smoking history and physical activity [29, 30]. The studies by Liu et al. in 2021 and 2022 each lost one point on the item ‘assessment of the outcome’ because the cognitive status of the participants was self-reported [6, 9]. The study by Zarifkar et al. lost two points on NOS comparability items, due to its failure to control for age, sex, and other factors [33]. The studies by Cohen et al. [27], Liu et al. in 2021 [6], Park et al. [29], and Taquet et al. in 2021 [31], lost one point on the NOS outcome items due to their failure to provide a follow-up period of at least 12 months for outcomes to occur.

### Overall pooled meta-analysis results from all 11 included studies

**Fig. 2:**
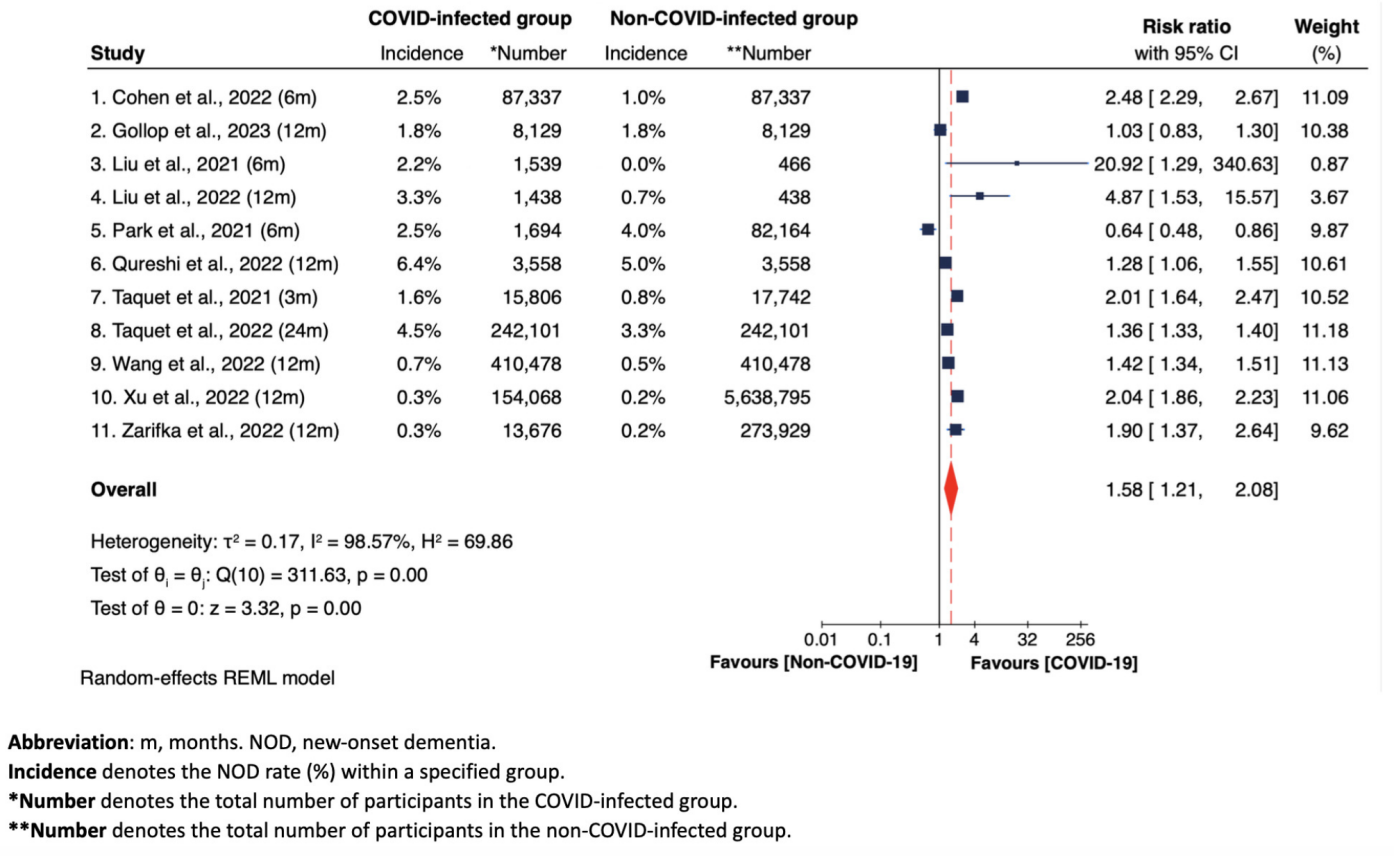
Forest plot of overall pooled meta-analysis of NOD risk between COVID-infected group and non-COVID-infected group across all 11 studies

In the main pooled analysis, the forest plot in Fig. 2 showed the differences in NOD risks between COVID-infected group and non-COVID-infected group across the 11 included studies. A random-effects REML model was used due to substantial heterogeneity. We did not distinguish between non-COVID-19 statuses, grouping together both healthy individuals and those with other types of respiratory infections as controls. The overall pooled analysis revealed a significant link between COVID-19 infection and increased risk for NOD in COVID-19 older adult survivors (RR = 1.58, 95% CI 1.21–2.08, *p* < 0.001; I^2^ = 98.57%, *p* < 0.001).

Among separate studies, nine out of 11 studies reported an increased risk for developing NOD in COVID-infected older adults, in comparison to their non-infected counterparts [6, 9, 12, 13, 27, 30–33]. Notably, compared to eight studies indicating a RR from 1.28 to 4.87, one study showed that COVID-19 infection led to a likelihood of developing NOD that was more than 20 times that of those uninfected (RR = 20.92, 95% CI 1.29-340.63) [6], albeit contributing minimally to the overall weight (0.87%). Zero dementia events in the non-COVID-infected group were reported in this study, which would theoretically result in an infinite risk ratio [6]. However, the statistical software addressed this challenge in meta-analyses by employing a continuity correction. For the study in question, this involved adding a nominal value of 0.5 to each cell of the 2 × 2 contingency table. This adjustment was designed to mitigate the computational difficulties posed by zero events and to allow for the estimation of an adjusted risk ratio [35]. The same approaches were applied across all meta-analyses, as necessary.

In contrast, one study suggested no significant difference in NOD risk between COVID-infected and non-infected groups (RR = 1.03, 95% CI 0.83–1.30) [28], while another study by Park et al. suggested a protective effect of COVID-19 infection against NOD risk (RR = 0.64, 95% CI 0.48–0.86) [29]. However, we noticed that this risk ratio (i.e., 0.64) was calculated by us without considering confounding covariates. In their original article [29], a consistently higher risk of NOD in COVID-infected individuals was observed across all age groups in their models adjusted for multiple covariates. Therefore, it can be inferred that, had adjustments for confounding factors been possible (which were not performed by us due to inaccessible relevant data), COVID-19 infection could still be associated with an increased NOD risk among older adult survivors in our analysis.

### Subgroup analyses

**Fig. 3:**
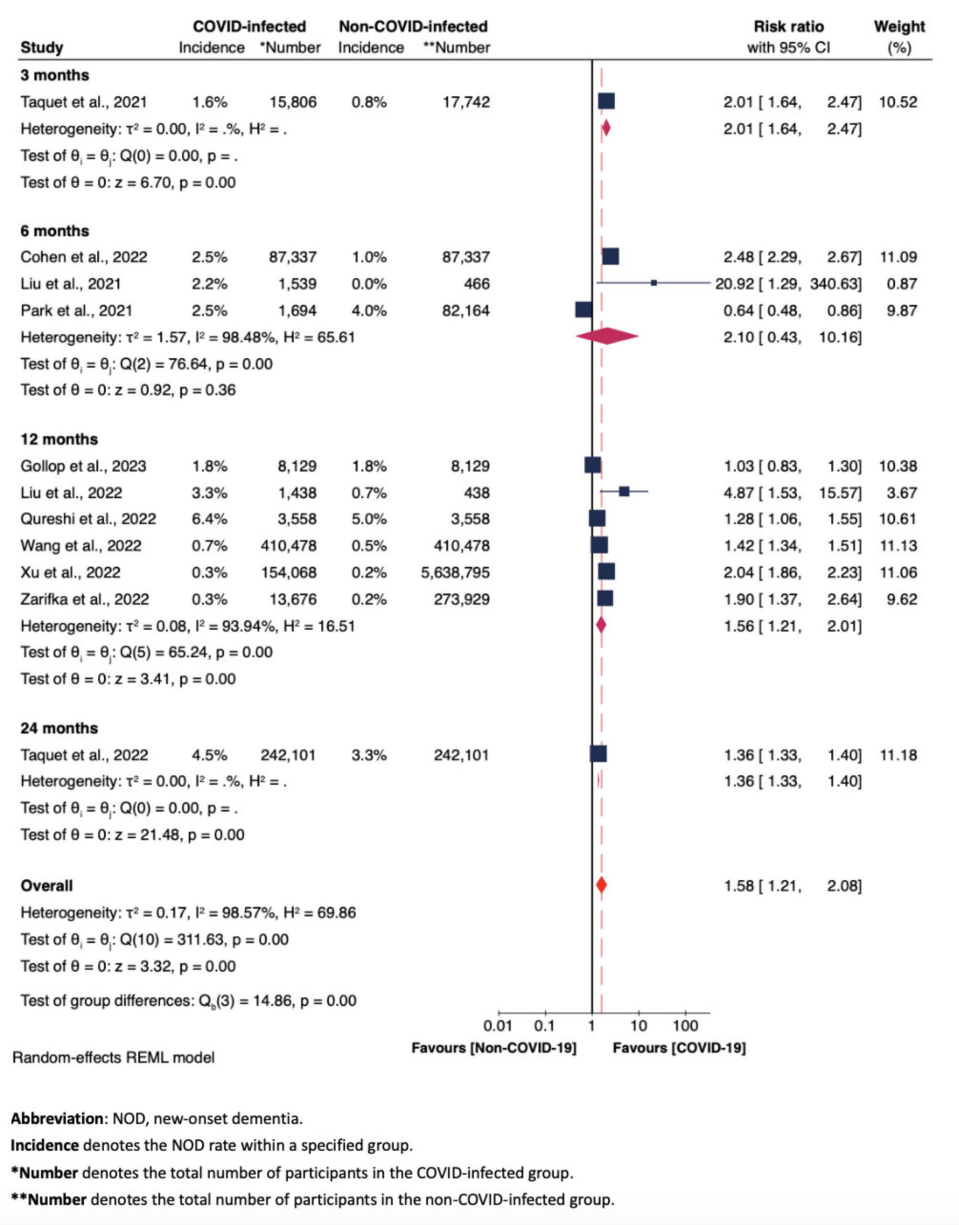
Forest plot of the meta-analysis of NOD risk between COVID-infected group and non-COVID-infected group at 3, 6, 12, 24 months, involving all 11 studies

**Fig. 4:**
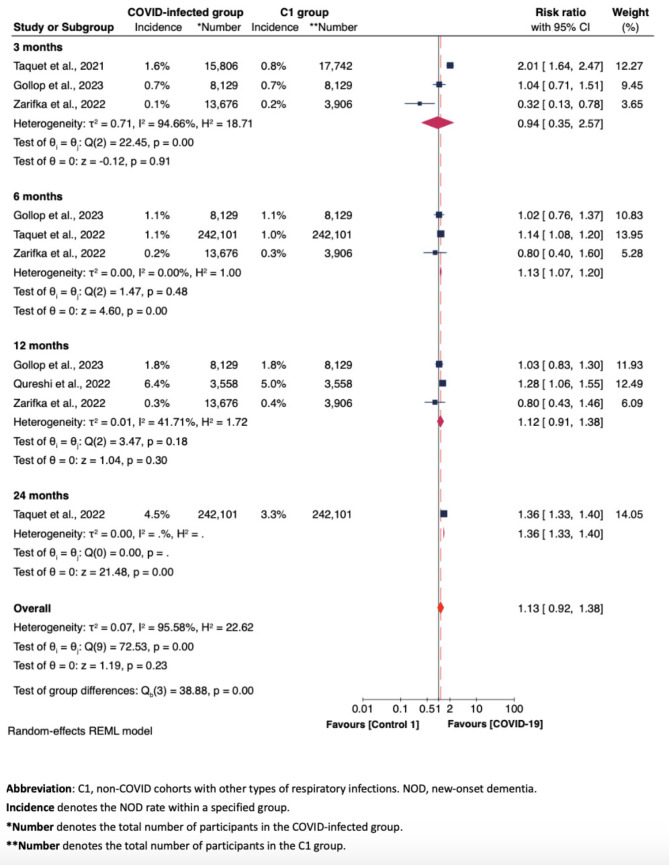
Forest plot of the meta-analysis of NOD risk between COVID-infected group and C1 group at 3, 6, 12, 24 months

**Fig. 5:**
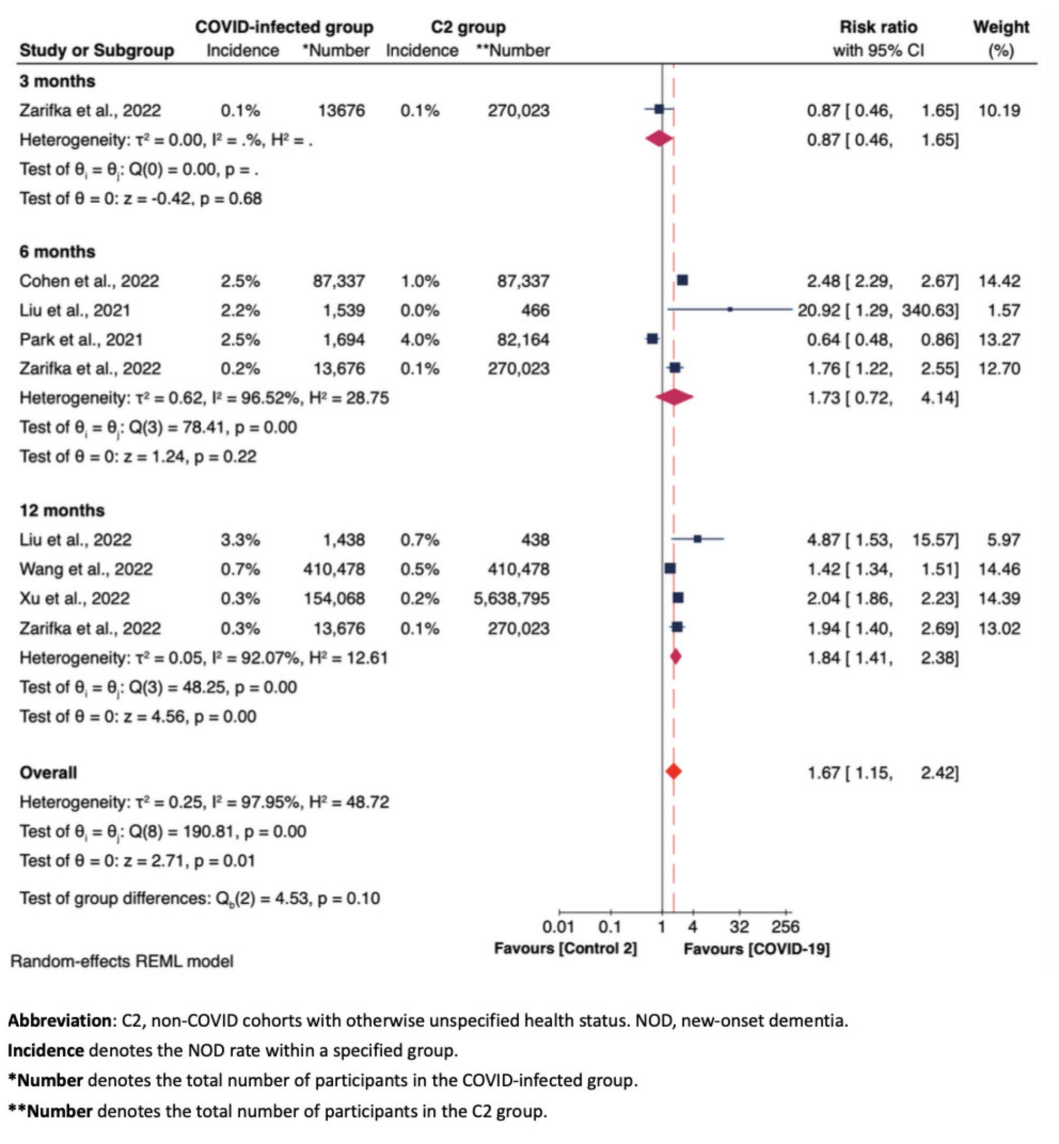
Forest plot of the meta-analysis of NOD risk between COVID-infected group and C2 group at 3, 6, 12, 24 months

Figures 3, 4 and 5, along with Supplementary Figures S1 to S5, display the results of subgroup analyses, examining: (i) NOD risk solely based on observational durations (i.e., at 3, 6, 12, 24 months) (Fig. 3); (ii) NOD risk based on COVID-19 infection status [infected vs. other respiratory infections (C1) (Fig. 4), and infected vs. uninfected (C2) (Fig. 5)]; (iii) risk of developing cognitive impairment in COVID-infected group compared to non-COVID-infected group (here C1 and C2 were grouped together), with cognitive impairment (including both CIND and dementia cases) as the measured outcome (Figure S1); (iv) NOD risk across three groups - those testing positive for COVID-19, those with other respiratory infections, and those testing negative for COVID-19 otherwise unspecified, specifically based on sex differences (Figure S2); and (v). NOD risk among COVID-19 patients, categorized by COVID-19 severity (Figure S3 and S4); and (vi) NOD risk between COVID-infected and non-COVID-infected groups among studies employing propensity-score matching approach (Figure S5). Most of these subgroup meta-analyses applied random-effects REML models due to substantial heterogeneity.

### NOD risk among COVID-infected and non-COVID-infected groups, based on observational periods

Figure 3 illustrates that, when examining pooled results from more than one individual study, the risk ratio at 12 months was significantly greater in the group infected with COVID-19 (RR = 1.56, 95% CI 1.21–2.01), closely aligning with the overall pooled risk ratio in Fig. 2 (RR = 1.58, 95% CI 1.21–2.08). Also, this subset exhibits reduced heterogeneity (I^2^ = 93.94%, Fig. 3) in comparison to the broader analysis of all 11 studies (I^2^ = 98.57%, Fig. 2). However, the increase in risk ratio at six months was not statistically significant (RR = 2.10, 95% CI 0.43–10.16). This lack of significance can be attributed to the outlier risk ratio of 0.64 reported by Park et al., which was a value before adjustment [29].

### NOD risk among COVID-Infected, non-COVID-infected otherwise unspecified, and non-COVID-infected with other respiratory infections groups across follow-up periods

Figure 4 shows no remarkable difference about NOD risk between the COVID-19 group and the non-COVID cohorts with other respiratory infections [C1 group] (overall RR = 1.13, 95% CI 0.92–1.38). Figure 5 shows a significantly increased risk for NOD in the COVID-19 group compared to the non-COVID cohorts with otherwise unspecified health statuses [C2 group] at 12 months post-COVID-19 (RR = 1.84, 95% CI 1.41–2.38). This increased risk was not evident at three months (RR = 0.87, 95% CI 0.46–1.65) or six months (RR = 1.73, 95% CI 0.72–4.14). Here the lack of statistical significance at six months can also be attributed to the result from Park et al. [29], which, once adjusted, could contribute to an overall significant increase in NOD risk at six months.

### Comparison of newly developed cognitive impairment risk between COVID-infected and non-COVID-infected groups

Figure S1 shows that, among the 3 studies which explored the risk of developing new-onset cognitive impairment between the COVID-Infected and the non-COVID-infected groups [6, 9, 27], a significant increased risk for NOD was observed in the COVID-infected group (overall RR = 1.93, 95% CI 1.52–2.43, *p* < 0.001; I^2^ = 79.04%, *p* < 0.001). In other words, cognitive impairment was nearly twice as likely in COVID-19 older adult survivors compared to those without COVID-19 infection.

### NOD risk based on sex in COVID-positive, other respiratory infection, and COVID-negative otherwise unspecified groups, separately

Notably, Figure S2 shows higher NOD risks for women on both the COVID-positive group (RR = 1.65, 95% CI 1.53–1.78, *p* < 0.001; I^2^ = 0.00%, *p* > 0.05) and COVID-negative otherwise unspecified control group (RR = 1.33, 95% CI 1.22–1.44, *p* < 0.001; I^2^ = 0.00%, *p* > 0.05), indicating that COVID-19 infection itself was not a major underlying factor making women more susceptible to developing NOD compared with men.

### NOD risk among COVID-infected patients, based on COVID-19 severity (inpatient vs. outpatient)

Both Figures S3 and S4 show significantly higher risks for NOD among COVID-infected older adult outpatients (RR = 1.91, 95% CI 1.06–3.45) and inpatients (RR = 3.06, 95% CI 2.78–3.37), as compared to COVID-negative older adults.

### NOD risk between COVID-infected and non-COVID-infected groups, based on studies with propensity score matching (PSM)

Figure S5 indicates that the only five studies using PSM reported an increased NOD risk in the COVID-infected group (Overall RR = 1.46, 95% CI 1.10–1.94) [12, 27, 28, 30, 32]. This increase in NOD risk is consistent with the findings from our main analysis, which includes all 11 studies (Overall RR = 1.58, Fig. 2).

### Overall heterogeneity and sensitivity analyses

We observed substantial heterogeneity among the 11 included studies in our main overall pooled meta-analysis in Fig. 2 (I^2^ = 98.57%, *p* < 0.001). Also, L’Abbé and Galbraith plots, as shown in Figure S6 and Figure S7, visually indicate the discrepancies among these studies. Contrary to expectations, the meta-regression results, as shown in Figure S8, suggest that covariates such as observational durations (3, 6, 12, 24 months), types of control groups (non-COVID cohorts otherwise unspecified vs. non-COVID cohorts with other types of respiratory infections), and dementia types assessed (all-cause dementia vs. AD) did not contribute to the variability among the 11 studies. The sensitivity analysis, as shown in Figure S9, suggests that the overall results remained consistent despite the removal of each individual study (with acceptable changes in effect size ranging from 0.09 to 0.12), indicating that the findings of our main meta-analysis in Fig. 2 were robust and not overly dependent on any single study.

### Publication bias

The contour-enhanced funnel plot, illustrated in Figure S10, visually indicates potential asymmetry, hinting at publication bias. Two imputed studies were strategically placed to mirror the asymmetrical gaps. However, the regression-based Egger’s test (*p* = 0.052) and the nonparametric rank correlation Begg’s test (*p* = 0.978) do not provide strong evidence of significant publication bias in our main overall pooled meta-analysis. Meanwhile, incorporating the two imputed studies into the analysis yields a revised pooled effect size for a total of 13 studies (RR = 1.48, 95% CI 1.12–1.96), which still does not markedly differ from the initial analysis (RR = 1.58, 95% CI 1.21–2.08). In addition, the average quality appraisal (NOS) score of 8.1 [standard deviation (SD) = 0.79] is suggestive of a good methodological quality of the 11 included studies. All included studies properly represent the target population, investigating the impact of COVID-19 on the NOD risk in older adults, with satisfactory sample sizes throughout.

## Discussion

In this review, we explored the association between COVID-19 infection and the risk of developing new-onset dementia (NOD) in older adults aged ≥ 60 years. Our overall pooled meta-analysis results indicated a heightened risk of developing NOD post-COVID-19 infection among older adults, in line with both our analytic results restricted to the studies employing propensity score matching and previous findings from a meta-analysis that covered all age groups [17]. This risk at 12 months post-COVID infection was closely aligned with our main analysis, but at six months, it was not statistically significant due to the outlier risk ratio of 0.64 from Park et al. [29], calculated before adjusting for covariates. After adjustments, an overall significantly increased NOD risk at six months is anticipated . Overall, we assume that remaining uninfected by COVID-19 may serve as a protective factor against the development of dementia over time among older adults. Based on the currently available follow-up periods post-infection, this assumption appears most robust at 12 months post-COVID-19 infection.

In our analysis of different types of control groups, we found that COVID-infected cohorts were at a significantly higher risk of developing NOD compared to those who were COVID-negative and had no specific health status mentioned, particularly at 12 months after infection. This increased risk was not seen at three and six months. The lack of significant findings at these earlier times may be because we included the original data from studies by Zarifka et al. and Park et al. [29, 33], which were not adjusted. Park et al. found a higher risk of NOD six months after COVID-19 when taking various factors into account [29], while Zarifka et al. argued that early comparisons (e.g., within three months) of NOD risk might not be reliable [33], especially in their study which did not adjust for multiple confounding factors. Hence, the comparison between the COVID-infected group and the COVID-negative otherwise unspecified group showed consistent results with our main analysis, highlighting a potential increase in NOD risk especially at 12 months following COVID-19 infection.

We found that COVID-19 and other respiratory infections pose a similar risk of leading to NOD. This may be attributed to the vulnerability of older adults and potentially similar neuroinflammatory impacts of other respiratory infections as observed with COVID-19 on the brain [36]. Evidence has suggested that seasonal influenza and SARS-CoV-2 are the most frequent viruses causing Acute Respiratory Distress Syndrome (ARDS) [37, 38], which could contribute to a high prevalence of cognitive impairment among older adults through mechanisms such as hypoxemia [39]. Also, the influenza virus could reach the central nervous system from the periphery [40, 41]. A prior study revealed that older adult patients hospitalized due to influenza, regardless of pneumonia presence, had a two to seven fold increase in the likelihood of developing AD, all-cause dementia, and vascular dementia [36]. Moreover, multiple studies have showed that influenza and pneumococcal vaccinations significantly reduce the risk of AD, particularly in older adults [36, 40, 42]. Chu et al. found that older adults hospitalized for bacterial pneumonia three or more times faced a 3.72-fold higher risk of developing dementia compared to those without a history of bacterial pneumonia. Those suffering from septicaemia as a consequence of bacterial pneumonia were at a tripled risk of developing dementia. The duration of ICU stay also influenced dementia risk: not being admitted to the ICU, staying in the ICU for one–six days, and staying for seven or more days were associated with 2.99, 2.80, and 2.42 times risks of dementia, respectively [43]. These results provided evidence that moderate-to-severe respiratory infections caused by microorganisms may significantly influence the NOD risk in this susceptible population. This is analogous to our observations of an elevated NOD risk in COVID-19 infected older adult inpatients compared to those uninfected by COVID-19. Nevertheless, despite substantial evidence highlighting the adverse effects of these microorganisms on cognitive functions, evidence in recent years on the NOD risk among outpatients with mild symptoms infected by these pathogens remain inconclusive, necessitating further research.

In our analyses, it was strikingly observed that severe COVID-19 infection increased the NOD risk by more than 20 times compared to those not infected, based on results by Liu et al. in 2021 [6]. This significant increase might be attributed to several reasons. Firstly, the classification of ‘severe COVID-19’ in the study by Liu et al. extended beyond mere hospitalization, adhering to the criteria set forth by the American Thoracic Society guidelines. This definition includes COVID-19 infected patients exhibiting any of the following: a respiratory rate exceeding 30 breaths per minute, severe respiratory distress, or an oxygen saturation below 90% in ambient air [6]. Secondly, whereas most included studies utilized ICD-10 codes to identify dementia diagnoses, Liu et al. opted for the TICS-40. It has been reported that ICD-10 codes achieve a sensitivity of 92.7% and a specificity of 98.9% in dementia diagnosis [44]. However, while Liu et al. noted that the Chinese version of the TICS-40 was validated by prior research [6], that study clarified its aim was not to confirm the TICS-40’s effectiveness as a screening instrument but rather to propose it as a suitable alternative to the Mini-Mental State Examination (MMSE) [45]. In addition to cognitive evaluations, considering factors such as neuroimaging and laboratory tests might be necessary [46, 47]. Therefore, the utility of the Chinese version of TICS-40 for dementia screening in older adults remains to be conclusively determined, indicating the need for further validation studies. Lastly, the initial COVID-19 variants investigated in Liu et al.‘s study may have posed a greater risk than later strains, particularly in the early stages of the pandemic when effective protective measures for cognitive functions were scarce [48, 49].

In the 11 studies reviewed, only one adjusted the potential influence of COVID-19 vaccination by including it as a covariate through propensity score matching [12]. To date, no scientific studies have conclusively demonstrated that coronavirus vaccinations directly affect the NOD risk. However, vaccinations for common diseases, such as influenza, have been shown to significantly lower the risk of dementia in the general population, even without considering previous infection histories. Moreover, more vaccinations correlated with a stronger protective effect against the development of dementia, outside the context of COVID-19 [50]. Wu et al. highlighted the absence of direct research on the effect of COVID-19 vaccinations on dementia risk but pointed out that considering the neurological complications associated with SARS-CoV-2, vaccination against COVID-19 might help reduce cognitive decline, offering some protection against NOD related to COVID-19 infection [50]. This review does not assert that vaccinations against COVID-19, influenza, or other diseases directly serve as a universal safeguard against dementia. Instead, we assume that vaccinations might alleviate the negative impact on cognitive function for those who contract COVID-19 or influenza. The direct preventive benefits of vaccinations against dementia in healthy individuals still require more substantial evidence.

This study presents multiple strengths. First and foremost, it is likely the first meta-analysis to comprehensively assess the impact of COVID-19 infection on the risk of developing NOD in older adults aged 60 years and older, across various time intervals. We provide evidence supporting the protective effects of avoiding COVID-19 and other respiratory infections in minimizing the risk of NOD. Secondly, many may argue that older adults experiencing pre-cognitive decline may exhibit reduced adherence to COVID-19 preventative measures, thus exposing an already cognitively vulnerable demographic to COVID-19 [51]. This exposure could potentially exaggerate the observed association between COVID-19 infection and NOD development, given the pre-existing cognitive vulnerabilities of the COVID-19 cohorts compared to healthier counterparts. Nonetheless, our analysis, restricted to the five studies using propensity score matching, indicated an increased risk of NOD consistent with the main analysis across 11 studies. This highlights the comparability and stability of our findings. This finding is important, because the 11 studies in our main analysis did not uniformly accounting for various confounders, especially participants’ pre-existing medical conditions and prior cognitive statuses. Thirdly, we employed multiple heterogeneity and sensitivity tests to confirm the robustness of our findings, indicating they are not excessively reliant on any single study. Meanwhile, the Trim-and-Fill method was used to address potential publication bias, which did not remarkably alter the main results, thereby affirming the reliability of our findings.

## Limitation and future directions

Our systematic review with meta-analysis encounters several limitations. Firstly, the number of available studies on this topic is relatively limited, and some subgroup analyses included only a small number of studies, which may reduce the statistical power and generalizability of the results. As more evidence emerges in the coming years, future research should aim to synthesize findings from a greater number of eligible studies, including those with longer follow-up periods and more diverse populations. Secondly, substantial statistical heterogeneity among the 11 studies was observed in the analytic results, potentially attributed to variations in the age groups of older adults explored, health-related baseline features of participants, characteristics of the non-COVID control groups, baseline time points for follow-up, duration of follow-up, diagnostic criteria for dementia, participant sample sizes, specific viral strains of COVID-19, and other undetectable factors. We extensively used random-effects REML models to minimize the impact of the heterogeneity we observed. Thirdly, most included studies in our review were retrospective, hindering our ability to infer causality or prospectively observe temporal changes. Fourthly, the results of the current meta‐analysis only provide evidence regarding the association between COVID-19 infection and NOD risk in our targeted population, and cannot be utilized to establish cause‐and‐effect relationships. Fifthly, our analyses were only able to be limited to evaluating the effects of different control groups, sex differences, and the severity of COVID-19 on the risk of developing NOD post-COVID-19 infection. We could not access data on other potential moderating factors. For instance, the lack of detailed data on NOD’s cumulative incidence across different age groups (i.e., 60–69, 70–79, 80–89 and ≥ 90 years) hindered our capacity to perform a meta-analysis evaluating the impact of COVID-19 on NOD risk among distinct age categories within older adults. Sixthly, the utilization of ICD-10 codes for NOD identification in most studies might introduce potential diagnostic variations due to diverse institutional criteria and coding practices [30].

We would like to highlight and discuss two additional significant limitations: 1). One included study indicated an increased NOD risk at 3 months post-COVID infection [31]. However, due to the prevalent occurrence of transient and reversible dementia-like symptoms—which could lead to false positive diagnoses of dementia in early phases post-COVID-19 infection—it is challenging to establish a clear association between COVID-19 infection and NOD risk during the early recovery phase (e.g., within 1–3 months post-infection) [28, 31, 51–53]. Meanwhile, we noticed that, in our review, only one study explored the association between COVID-19 and NOD at 24 months post-COVID infection, with others spanning 3–12 months. This study also showed a higher NOD risk post-COVID infection [12]. Considering dementia’s slow progression and the potential for long-COVID to cause lasting and progressive cognitive impact [40], a 3–12 month observation may not suffice, potentially skewing results. Research with longer observational periods is necessary to better understand COVID-19’s long-term neurological impacts. Nonetheless, considering dementia’s irreversible nature and its significant impact on individuals, families, and society, we believe that providing evidence early, despite these challenges, is more beneficial than solely focusing on determining the precise risks of COVID-19 precipitating NOD through rigorous prospective studies. This is why conducting our study at this moment is crucial; 2). we aimed to include evidence on comorbidities and detailed hospitalization data (e.g., receiving oxygen therapy, ICU admission) in subgroup analyses but were constrained by the lack of sufficient data among older adults with and without NOD in original studies, limiting our ability to conduct a thorough meta-analysis on these covariates’ effects. However, our analysis, when restricted to studies using propensity score matching (PSM), yielded a result consistent with our main findings, despite the relatively small number of studies employing the PSM method (*n* = 5).

These limitations in our review emphasize areas for future directions, when addressable. Also, potentially varied risks of different types of dementia related to COVID-infection, the effects of multiple COVID infections and vaccination status, and the development of prevention and early rehabilitation strategies may also hold value for future research.

## Conclusions

We found that, compared to all non-COVID controls, COVID-19 infection is significantly associated with an increased risk of developing new-onset dementia in older adults aged 60 years and above, with the most robust evidence for this association observed at 12 months post-infection during the recovery stages, based on currently available follow-up observational periods from prior research. Despite this, the extent to which COVID-19 may elevate the risk of new-onset dementia still warrants further investigation.

## Electronic supplementary material

Below is the link to the electronic supplementary material.

Supplementary Material 1

## Data Availability

All data relevant to the current study are included in this manuscript or available from the supplementary files uploaded.
