# Supplementary material for: Association between COVID-19 infection and new-onset dementia in older adults: a systematic review and meta-analysis
Source: BMC Geriatr. 2024 Dec 15;24:940. doi: 10.1186/s12877-024-05538-5 (PMC11646349; doi:10.1186/s12877-024-05538-5)
Supplement: Supplementary file 1 — Supplementary Material 1 [file 12877_2024_5538_MOESM1_ESM.docx]

**Title: Association Between COVID-19 Infection and New-Onset Dementia in Older Adults: A Systematic Review and Meta-Analysis**

**Supplementary Materials**

**Contents**

[**Table S1.** PRISMA 2020 Checklist. 2](#_Toc161028697)

[**Table S2.** Literature search strategy. 7](#_Toc161028698)

[**Table S3.** A list of partial studies along with reasons for their exclusion. 11](#_Toc161028699)

[**Table S4(a).** Quality assessment of included studies via the Newcastle-Ottawa Scale (NOS) for cohort studies. 14](#_Toc161028700)

[**Table S4(b).** Quality assessment of an included study via the Newcastle-Ottawa Scale (NOS), adapted for cross-sectional studies. 15](#_Toc161028701)

[**Figure S1.** Forest plot of the meta-analysis of the risk of new-onset cognitive impairments (including both CIND and dementia) between COVID-infected group and non-COVID-infected group. 16](#_Toc161028702)

[**Figure S2.** Forest plot of the subgroup meta-analyses of the new-onset dementia risk in COVID-positive group, other respiratory infection group, and COVID-negative otherwise unspecified group, separately, based on sex (i.e., male vs. female). 17](#_Toc161028703)

[**Figure S3.** Forest plot of the meta-analysis of the new-onset dementia risk between non-COVID-hospitalized (i.e., outpatient) and COVID-negative groups. 18](#_Toc161028704)

[**Figure S4.** Forest plot of the meta-analysis of the new-onset dementia risk between COVID-hospitalized (i.e., inpatient) and COVID-negative groups. 19](#_Toc161028705)

[**Figure S5.** Forest plot of the meta-analysis of the new-onset dementia risk between COVID-infected group and non-COVID-infected group, across five studies employing Propensity Score Matching (PSM). 20](#_Toc161028706)

[**Figure S6.** L'Abbé plot. 21](#_Toc161028707)

[**Figure S7.** Galbraith plot. 22](#_Toc161028708)

[**Figure S8.** Random-effects meta-regression model. 23](#_Toc161028709)

[**Figure S9.** Sensitivity analysis. 24](#_Toc161028710)

[**Figure S10.** Contour-enhanced funnel plot. 25](#_Toc161028711)

# **Table S1.** PRISMA 2020 Checklist.

| **Section and Topic** | **Item #** | **Checklist item** | **Location where item is reported** |
| --- | --- | --- | --- |
| **TITLE** | | |  |
| Title | 1 | Identify the report as a systematic review. | See title |
| **ABSTRACT** | | |  |
| Abstract | 2 | See the PRISMA 2020 for Abstracts checklist. | Section 2.0 |
| **INTRODUCTION** | | |  |
| Rationale | 3 | Describe the rationale for the review in the context of existing knowledge. | Section 1.0 |
| Objectives | 4 | Provide an explicit statement of the objective(s) or question(s) the review addresses. | Section 1.0 |
| **METHODS** | | |  |
| Eligibility criteria | 5 | Specify the inclusion and exclusion criteria for the review and how studies were grouped for the syntheses. | Section 2.1 |
| Information sources | 6 | Specify all databases, registers, websites, organisations, reference lists and other sources searched or consulted to identify studies. Specify the date when each source was last searched or consulted. | Section 2.1 |
| Search strategy | 7 | Present the full search strategies for all databases, registers and websites, including any filters and limits used. | Section 2.1 |
| Selection process | 8 | Specify the methods used to decide whether a study met the inclusion criteria of the review, including how many reviewers screened each record and each report retrieved, whether they worked independently, and if applicable, details of automation tools used in the process. | Section 2.2 |
| Data collection process | 9 | Specify the methods used to collect data from reports, including how many reviewers collected data from each report, whether they worked independently, any processes for obtaining or confirming data from study investigators, and if applicable, details of automation tools used in the process. | Section 2.2 |
| Data items | 10a | List and define all outcomes for which data were sought. Specify whether all results that were compatible with each outcome domain in each study were sought (e.g. for all measures, time points, analyses), and if not, the methods used to decide which results to collect. | Section 2.2 |
|  | 10b | List and define all other variables for which data were sought (e.g. participant and intervention characteristics, funding sources). Describe any assumptions made about any missing or unclear information. | Section 2.2 |
| Study risk of bias assessment | 11 | Specify the methods used to assess risk of bias in the included studies, including details of the tool(s) used, how many reviewers assessed each study and whether they worked independently, and if applicable, details of automation tools used in the process. | Section 2.3 |
| Effect measures | 12 | Specify for each outcome the effect measure(s) (e.g. risk ratio, mean difference) used in the synthesis or presentation of results. | Section 2.3 |
| Synthesis methods | 13a | Describe the processes used to decide which studies were eligible for each synthesis (e.g. tabulating the study intervention characteristics and comparing against the planned groups for each synthesis (item #5)). | Section 2.3 |
|  | 13b | Describe any methods required to prepare the data for presentation or synthesis, such as handling of missing summary statistics, or data conversions. | N/A |
|  | 13c | Describe any methods used to tabulate or visually display results of individual studies and syntheses. | Section 2.3 |
|  | 13d | Describe any methods used to synthesize results and provide a rationale for the choice(s). If meta-analysis was performed, describe the model(s), method(s) to identify the presence and extent of statistical heterogeneity, and software package(s) used. | Section 2.3 |
|  | 13e | Describe any methods used to explore possible causes of heterogeneity among study results (e.g. subgroup analysis, meta-regression). | Section 2.3 |
|  | 13f | Describe any sensitivity analyses conducted to assess robustness of the synthesized results. | Section 2.3 |
| Reporting bias assessment | 14 | Describe any methods used to assess risk of bias due to missing results in a synthesis (arising from reporting biases). | N/A |
| Certainty assessment | 15 | Describe any methods used to assess certainty (or confidence) in the body of evidence for an outcome. | N/A |
| **RESULTS** | | |  |
| Study selection | 16a | Describe the results of the search and selection process, from the number of records identified in the search to the number of studies included in the review, ideally using a flow diagram. | Section 3.1 & Figure 1 |
|  | 16b | Cite studies that might appear to meet the inclusion criteria, but which were excluded, and explain why they were excluded. | Figure 1 |
| Study characteristics | 17 | Cite each included study and present its characteristics. | Section 3.1 & Table 1 |
| Risk of bias in studies | 18 | Present assessments of risk of bias for each included study. | Table S4(a) & Table S4(b) |
| Results of individual studies | 19 | For all outcomes, present, for each study: (a) summary statistics for each group (where appropriate) and (b) an effect estimate and its precision (e.g. confidence/credible interval), ideally using structured tables or plots. | Figures 2-5,  Figures S1-S5 |
| Results of syntheses | 20a | For each synthesis, briefly summarise the characteristics and risk of bias among contributing studies. | Section 3 |
|  | 20b | Present results of all statistical syntheses conducted. If meta-analysis was done, present for each the summary estimate and its precision (e.g. confidence/credible interval) and measures of statistical heterogeneity. If comparing groups, describe the direction of the effect. | Section 3 |
|  | 20c | Present results of all investigations of possible causes of heterogeneity among study results. | Figures S6-S8 |
|  | 20d | Present results of all sensitivity analyses conducted to assess the robustness of the synthesized results. | Figure S9 |
| Reporting biases | 21 | Present assessments of risk of bias due to missing results (arising from reporting biases) for each synthesis assessed. | Table S4(a) & Table S4(b) |
| Certainty of evidence | 22 | Present assessments of certainty (or confidence) in the body of evidence for each outcome assessed. | N/A |
| **DISCUSSION** | | |  |
| Discussion | 23a | Provide a general interpretation of the results in the context of other evidence. | Section 4 |
|  | 23b | Discuss any limitations of the evidence included in the review. | Section 5 |
|  | 23c | Discuss any limitations of the review processes used. | Section 5 |
|  | 23d | Discuss implications of the results for practice, policy, and future research. | Sections 5 & 6 |
| **OTHER INFORMATION** | | |  |
| Registration and protocol | 24a | Provide registration information for the review, including register name and registration number, or state that the review was not registered. | Section 2.0 |
|  | 24b | Indicate where the review protocol can be accessed, or state that a protocol was not prepared. | Section 2.0 |
|  | 24c | Describe and explain any amendments to information provided at registration or in the protocol. | N/A |
| Support | 25 | Describe sources of financial or non-financial support for the review, and the role of the funders or sponsors in the review. | line 633-639 |
| Competing interests | 26 | Declare any competing interests of review authors. | line 626-627 |
| Availability of data, code and other materials | 27 | Report which of the following are publicly available and where they can be found: template data collection forms; data extracted from included studies; data used for all analyses; analytic code; any other materials used in the review. | line 629-631 |

| **Section and Topic** | **Item #** | **Checklist item** | **Reported (Yes/No)** |
| --- | --- | --- | --- |
| **TITLE** | | |  |
| Title | 1 | Identify the report as a systematic review and/or meta-analysis. | Yes |
| **BACKGROUND** | | |  |
| Objectives | 2 | Provide an explicit statement of the main objective(s) or question(s) the review addresses. | Yes |
| **METHODS** | | |  |
| Eligibility criteria | 3 | Specify the inclusion and exclusion criteria for the review. | Yes |
| Information sources | 4 | Specify the information sources (e.g. databases, registers) used to identify studies and the date when each was last searched. | Yes |
| Risk of bias | 5 | Specify the methods used to assess risk of bias in the included studies. | Yes |
| Synthesis of results | 6 | Specify the methods used to present and synthesise results. | Yes |
| **RESULTS** | | |  |
| Included studies | 7 | Give the total number of included studies and participants and summarise relevant characteristics of studies. | Yes |
| Synthesis of results | 8 | Present results for main outcomes, preferably indicating the number of included studies and participants for each. If meta-analysis was done, report the summary estimate and confidence/credible interval. If comparing groups, indicate the direction of the effect (i.e. which group is favoured). | Yes |
| **DISCUSSION** | | |  |
| Limitations of evidence | 9 | Provide a brief summary of the limitations of the evidence included in the review (e.g. study risk of bias, inconsistency and imprecision). | Yes |
| Interpretation | 10 | Provide a general interpretation of the results and important implications. | Yes |
| **OTHER** | | |  |
| Funding | 11 | Specify the primary source of funding for the review. | No |
| Registration | 12 | Provide the register name and registration number. | Yes |

*From:*  Page MJ, McKenzie JE, Bossuyt PM, Boutron I, Hoffmann TC, Mulrow CD, et al. The PRISMA 2020 statement: an updated guideline for reporting systematic reviews. BMJ 2021;372:n71. doi: 10.1136/bmj.n71

For more information, visit: <http://www.prisma-statement.org/>

# **Table S2.** Literature search strategy.

- **PubMed search details:**

| Search | Search term |
| --- | --- |
| #1 | ((((((COVID-19) OR (SARS-CoV-2)) OR (coronavirus)) OR (pandemic)) OR (post-COVID syndrome)) OR (long COVID)) OR (chronic COVID) |
| #2 | (((((Alzheimer's Disease) OR (dementia)) OR (neurodegenerative disorder)) OR (neurodegeneration)) OR (neurological sequelae)) OR (brain health) |
| #3 | (((((older adult) OR (elderly)) OR (geriatric)) OR (senior population)) OR (aging)) OR (ageing) |
| #4 | #1 AND #2 AND #3 |

***2834 results for***

**((((((((COVID-19) OR (SARS-CoV-2)) OR (coronavirus)) OR (pandemic)) OR (post-COVID syndrome)) OR (long COVID)) OR (chronic COVID)) AND ((((((Alzheimer's Disease) OR (dementia)) OR (neurodegenerative disorder)) OR (neurodegeneration)) OR (neurological sequelae)) OR (brain health))) AND ((((((older adult) OR (elderly)) OR (geriatric)) OR (senior population)) OR (aging)) OR (ageing))**

("covid 19"[All Fields] OR "covid 19"[MeSH Terms] OR "covid 19 vaccines"[All Fields] OR "covid 19 vaccines"[MeSH Terms] OR "covid 19 serotherapy"[All Fields] OR "covid 19 nucleic acid testing"[All Fields] OR "covid 19 nucleic acid testing"[MeSH Terms] OR "covid 19 serological testing"[All Fields] OR "covid 19 serological testing"[MeSH Terms] OR "covid 19 testing"[All Fields] OR "covid 19 testing"[MeSH Terms] OR "sars cov 2"[All Fields] OR "sars cov 2"[MeSH Terms] OR "severe acute respiratory syndrome coronavirus 2"[All Fields] OR "ncov"[All Fields] OR "2019 ncov"[All Fields] OR (("coronavirus"[MeSH Terms] OR "coronavirus"[All Fields] OR "cov"[All Fields]) AND 2019/11/01:3000/12/31[Date - Publication]) OR ("sars cov 2"[MeSH Terms] OR "sars cov 2"[All Fields] OR "sars cov 2"[All Fields]) OR ("coronavirus"[MeSH Terms] OR "coronavirus"[All Fields] OR "coronaviruses"[All Fields]) OR ("pandemic s"[All Fields] OR "pandemically"[All Fields] OR "pandemicity"[All Fields] OR "pandemics"[MeSH Terms] OR "pandemics"[All Fields] OR "pandemic"[All Fields]) OR ("post-COVID"[All Fields] AND ("syndrom"[All Fields] OR "syndromal"[All Fields] OR "syndromally"[All Fields] OR "syndrome"[MeSH Terms] OR "syndrome"[All Fields] OR "syndromes"[All Fields] OR "syndrome s"[All Fields] OR "syndromic"[All Fields] OR "syndroms"[All Fields])) OR ("post acute covid 19 syndrome"[MeSH Terms] OR ("post acute"[All Fields] AND "covid 19"[All Fields] AND "syndrome"[All Fields]) OR "post acute covid 19 syndrome"[All Fields] OR ("long"[All Fields] AND "covid"[All Fields]) OR "long covid"[All Fields]) OR (("chronic"[All Fields] OR "chronical"[All Fields] OR "chronically"[All Fields] OR "chronicities"[All Fields] OR "chronicity"[All Fields] OR "chronicization"[All Fields] OR "chronics"[All Fields]) AND ("sars cov 2"[MeSH Terms] OR "sars cov 2"[All Fields] OR "covid"[All Fields] OR "covid 19"[MeSH Terms] OR "covid 19"[All Fields]))) AND ("alzheimer disease"[MeSH Terms] OR ("alzheimer"[All Fields] AND "disease"[All Fields]) OR "alzheimer disease"[All Fields] OR ("alzheimer s"[All Fields] AND "disease"[All Fields]) OR "alzheimer s disease"[All Fields] OR ("dementia"[MeSH Terms] OR "dementia"[All Fields] OR "dementias"[All Fields] OR "dementia s"[All Fields]) OR ("neurodegenerative diseases"[MeSH Terms] OR ("neurodegenerative"[All Fields] AND "diseases"[All Fields]) OR "neurodegenerative diseases"[All Fields] OR ("neurodegenerative"[All Fields] AND "disorder"[All Fields]) OR "neurodegenerative disorder"[All Fields]) OR ("nerve degeneration"[MeSH Terms] OR ("nerve"[All Fields] AND "degeneration"[All Fields]) OR "nerve degeneration"[All Fields] OR "neurodegeneration"[All Fields] OR "neurodegenerating"[All Fields] OR "neurodegenerations"[All Fields]) OR (("neurologic"[All Fields] OR "neurological"[All Fields] OR "neurologically"[All Fields]) AND ("complications"[MeSH Subheading] OR "complications"[All Fields] OR "sequelae"[All Fields] OR "sequela"[All Fields] OR "sequelaes"[All Fields] OR "sequelas"[All Fields])) OR health, brain[Investigator]) AND ("aged"[MeSH Terms] OR "aged"[All Fields] OR ("older"[All Fields] AND "adult"[All Fields]) OR "older adult"[All Fields] OR ("aged"[MeSH Terms] OR "aged"[All Fields] OR "elderly"[All Fields] OR "elderlies"[All Fields] OR "elderly s"[All Fields] OR "elderlys"[All Fields]) OR ("geriatric"[All Fields] OR "geriatrics"[MeSH Terms] OR "geriatrics"[All Fields]) OR (("senior"[All Fields] OR "seniorities"[All Fields] OR "seniority"[All Fields] OR "seniors"[All Fields]) AND ("populate"[All Fields] OR "populated"[All Fields] OR "populates"[All Fields] OR "populating"[All Fields] OR "population"[MeSH Terms] OR "population"[All Fields] OR "population groups"[MeSH Terms] OR ("population"[All Fields] AND "groups"[All Fields]) OR "population groups"[All Fields] OR "populations"[All Fields] OR "population s"[All Fields] OR "populational"[All Fields] OR "populous"[All Fields])) OR ("aging"[MeSH Terms] OR "aging"[All Fields] OR "ageing"[All Fields]) OR ("aging"[MeSH Terms] OR "aging"[All Fields] OR "ageing"[All Fields]))

**Translations**

**COVID-19:** ("COVID-19" OR "COVID-19"[MeSH Terms] OR "COVID-19 Vaccines" OR "COVID-19 Vaccines"[MeSH Terms] OR "COVID-19 serotherapy" OR "COVID-19 serotherapy"[Supplementary Concept] OR "COVID-19 Nucleic Acid Testing" OR "covid-19 nucleic acid testing"[MeSH Terms] OR "COVID-19 Serological Testing" OR "covid-19 serological testing"[MeSH Terms] OR "COVID-19 Testing" OR "covid-19 testing"[MeSH Terms] OR "SARS-CoV-2" OR "sars-cov-2"[MeSH Terms] OR "Severe Acute Respiratory Syndrome Coronavirus 2" OR "NCOV" OR "2019 NCOV" OR (("coronavirus"[MeSH Terms] OR "coronavirus" OR "COV") AND 2019/11/01[PDAT] : 3000/12/31[PDAT]))

**SARS-CoV-2:** "sars-cov-2"[MeSH Terms] OR "sars-cov-2"[All Fields] OR "sars cov 2"[All Fields]

**coronavirus:** "coronavirus"[MeSH Terms] OR "coronavirus"[All Fields] OR "coronaviruses"[All Fields]

**pandemic:** "pandemic's"[All Fields] OR "pandemically"[All Fields] OR "pandemicity"[All Fields] OR "pandemics"[MeSH Terms] OR "pandemics"[All Fields] OR "pandemic"[All Fields]

**syndrome:** "syndrom"[All Fields] OR "syndromal"[All Fields] OR "syndromally"[All Fields] OR "syndrome"[MeSH Terms] OR "syndrome"[All Fields] OR "syndromes"[All Fields] OR "syndrome's"[All Fields] OR "syndromic"[All Fields] OR "syndroms"[All Fields]

**long COVID:** "post-acute covid-19 syndrome"[MeSH Terms] OR ("post-acute"[All Fields] AND "covid-19"[All Fields] AND "syndrome"[All Fields]) OR "post-acute covid-19 syndrome"[All Fields] OR ("long"[All Fields] AND "covid"[All Fields]) OR "long covid"[All Fields]

**chronic:** "chronic"[All Fields] OR "chronical"[All Fields] OR "chronically"[All Fields] OR "chronicities"[All Fields] OR "chronicity"[All Fields] OR "chronicization"[All Fields] OR "chronics"[All Fields]

**COVID:** "sars-cov-2"[MeSH Terms] OR "sars-cov-2"[All Fields] OR "covid"[All Fields] OR "covid-19"[MeSH Terms] OR "covid-19"[All Fields]

**Alzheimer's Disease:** "alzheimer disease"[MeSH Terms] OR ("alzheimer"[All Fields] AND "disease"[All Fields]) OR "alzheimer disease"[All Fields] OR ("alzheimer's"[All Fields] AND "disease"[All Fields]) OR "alzheimer's disease"[All Fields]

**dementia:** "dementia"[MeSH Terms] OR "dementia"[All Fields] OR "dementias"[All Fields] OR "dementia's"[All Fields]

**neurodegenerative disorder:** "neurodegenerative diseases"[MeSH Terms] OR ("neurodegenerative"[All Fields] AND "diseases"[All Fields]) OR "neurodegenerative diseases"[All Fields] OR ("neurodegenerative"[All Fields] AND "disorder"[All Fields]) OR "neurodegenerative disorder"[All Fields]

**neurodegeneration:** "nerve degeneration"[MeSH Terms] OR ("nerve"[All Fields] AND "degeneration"[All Fields]) OR "nerve degeneration"[All Fields] OR "neurodegeneration"[All Fields] OR "neurodegenerating"[All Fields] OR "neurodegenerations"[All Fields]

**neurological:** "neurologic"[All Fields] OR "neurological"[All Fields] OR "neurologically"[All Fields]

**sequelae:** "complications"[Subheading] OR "complications"[All Fields] OR "sequelae"[All Fields] OR "sequela"[All Fields] OR "sequelaes"[All Fields] OR "sequelas"[All Fields]

**brain health:** Health, Brain[Full Investigator Name]

**older adult:** "aged"[MeSH Terms] OR "aged"[All Fields] OR ("older"[All Fields] AND "adult"[All Fields]) OR "older adult"[All Fields]

**elderly:** "aged"[MeSH Terms] OR "aged"[All Fields] OR "elderly"[All Fields] OR "elderlies"[All Fields] OR "elderly's"[All Fields] OR "elderlys"[All Fields]

**geriatric:** "geriatric"[All Fields] OR "geriatrics"[MeSH Terms] OR "geriatrics"[All Fields]

**senior:** "senior"[All Fields] OR "seniorities"[All Fields] OR "seniority"[All Fields] OR "seniors"[All Fields]

**population:** "populate"[All Fields] OR "populated"[All Fields] OR "populates"[All Fields] OR "populating"[All Fields] OR "population"[MeSH Terms] OR "population"[All Fields] OR "population groups"[MeSH Terms] OR ("population"[All Fields] AND "groups"[All Fields]) OR "population groups"[All Fields] OR "populations"[All Fields] OR "population's"[All Fields] OR "populational"[All Fields] OR "populations's"[All Fields] OR "populous"[All Fields]

**aging:** "aging"[MeSH Terms] OR "aging"[All Fields] OR "ageing"[All Fields]

**ageing:** "aging"[MeSH Terms] OR "aging"[All Fields] OR "ageing"[All Fields]

- **APA PsycINFO search details:**

***434 results for***

(covid-19 or coronavirus or 2019-ncov or sars-cov-2 or cov-19 or pandemic) [TX All Text] AND (alzheimer's disease or alzheimers or alzheimer or alzheimer's or dementia or neurodegenerative disorder) [TX All Text] AND (older adults or elderly or seniors or geriatrics or ageing) [TX All Text]

- **Scopus search details:**

***3460 results for***

("COVID-19" OR "SARS-CoV-2" OR "coronavirus" OR "pandemic" OR "post-COVID syndrome" OR "long COVID" OR "chronic COVID”) [Article title, Abstract, Keywords] AND ("Alzheimer's Disease" OR "dementia" OR "neurodegenerative disorder" OR "neurodegeneration" OR "neurological sequelae" OR "brain health”) [Article title, Abstract, Keywords] AND ("older adult" OR "elderly" OR "geriatric" OR "senior population" OR "aging" OR “ageing”) [All fields]

- **MedRxiv preprint server search details:**

***223 Results for***

"COVID AND dementia AND older adults [Search Terms & Keywords]”

(64 in Geriatric Medicine section; 101 in Neurology section; 58 in Psychiatry and Clinical Psychology section)

- **PQDT Global search details:**

***389 results for terms***

“covid AND dementia AND older adults” [Source type: Dissertations & Theses}.

# **Table S3.** A list of partial studies along with reasons for their exclusion.

|  | Excluded studies | Exclusion reason |
| --- | --- | --- |
| 1. | van den Borst, B., Peters, J. B., Brink, M., Schoon, Y., Bleeker-Rovers, C. P., Schers, H., van Hees, H. W. H., van Helvoort, H., van den Boogaard, M., van der Hoeven, H., Reijers, M. H., Prokop, M., Vercoulen, J., & van den Heuvel, M. (2021). Comprehensive health assessment 3 months after recovery from COVID-19. ***Clinical Infectious Diseases : An Official Publication of the Infectious Diseases Society of America, 73***(5), e1089–e1098. | **New-onset dementia is not an outcome** |
| 2. | Dubey, S., Das, S., Ghosh, R., Dubey, M. J., Chakraborty, A. P., Roy, D., Das, G., Dutta, A., Santra, A., Sengupta, S., & Benito-León, J. (2023). The effects of SARS-CoV-2 infection on the cognitive functioning of patients with pre-existing dementia. ***Journal of Alzheimer's Disease Reports, 7***(1), 119–128. | **New-onset dementia is not an outcome** |
| 3. | Ayoubkhani, D., Khunti, K., Nafilyan, V., Maddox, T., Humberstone, B., Diamond, I., & Banerjee, A. (2021). Post-covid syndrome in individuals admitted to hospital with COVID-19: Retrospective cohort study. ***BMJ, 372***, n693. | **New-onset dementia is not an outcome** |
| 4. | Ghaffari, M., Ansari, H., Beladimoghadam, N., Aghamiri, S. H., Haghighi, M., Nabavi, M., Mansouri, B., Mehrpour, M., Assarzadegan, F., Hesami, O., Sedaghat, M., Farahbakhsh, M., & Lima, B. S. (2021). Neurological features and outcome in COVID-19: Dementia can predict severe disease. ***Journal of Neurovirology, 27***(1), 86–93 | **New-onset dementia is not an outcome** |
| 5. | Tondo, G., Sarasso, B., Serra, P., Tesser, F., & Comi, C. (2021). The impact of the COVID-19 pandemic on the cognition of people with dementia. ***International Journal of Environmental Research and Public Health, 18***(8), 4285. | **New-onset dementia is not an outcome** |
| 6. | Gan, J., Liu, S., Wu, H., Chen, Z., Fei, M., Xu, J., Dou, Y., Wang, X., & Ji, Y. (2021). The impact of the COVID-19 pandemic on Alzheimer's Disease and other dementias. ***Frontiers in Psychiatry, 12***, 703481. | **COVID-19 infection was not investigated in participants** |
| 7. | Davis, H. E., Assaf, G. S., McCorkell, L., Wei, H., Low, R. J., Re'em, Y., Redfield, S., Austin, J. P., & Akrami, A. (2021). Characterizing long COVID in an international cohort: 7 months of symptoms and their impact. ***EClinicalMedicine, 38***, 101019. | **New-onset dementia is not an outcome** |
| 8. | Al-Aly, Z., Bowe, B., & Xie, Y. (2022). Long COVID after breakthrough SARS-CoV-2 infection. ***Nature Medicine, 28***(7), 1461–1467. | **Information on the incidence of dementia among older adults was not available** |
| 9. | Daugherty, S. E., Guo, Y., Heath, K., Dasmariñas, M. C., Jubilo, K. G., Samranvedhya, J., Lipsitch, M., & Cohen, K. (2021). Risk of clinical sequelae after the acute phase of SARS-CoV-2 infection: Retrospective cohort study. ***BMJ, 373***, n1098. | **Information on the incidence of dementia among older adults was not available** |
| 10. | Wang, Q., Davis, P. B., Gurney, M. E., & Xu, R. (2021). COVID-19 and dementia: Analyses of risk, disparity, and outcomes from electronic health records in the US. ***Alzheimer's & Dementia : The Journal of the Alzheimer's Association, 17***(8), 1297–1306. | **New-onset dementia is not an outcome** |
| 11. | Huang, C., Huang, L., Wang, Y., Li, X., Ren, L., Gu, X., Kang, L., Guo, L., Liu, M., Zhou, X., Luo, J., Huang, Z., Tu, S., Zhao, Y., Chen, L., Xu, D., Li, Y., Li, C., Peng, L., Li, Y., … Cao, B. (2021). 6-month consequences of COVID-19 in patients discharged from hospital: A cohort study. ***Lancet (London, England), 397***(10270), 220–232. | **New-onset dementia is not an outcome** |
| 12. | Taquet, M., Dercon, Q., Luciano, S., Geddes, J. R., Husain, M., & Harrison, P. J. (2021). Incidence, co-occurrence, and evolution of long-COVID features: A 6-month retrospective cohort study of 273,618 survivors of COVID-19. ***PLoS Medicine, 18***(9), e1003773. | **New-onset dementia is not an outcome** |
| 13. | Taquet, M., Geddes, J. R., Husain, M., Luciano, S., & Harrison, P. J. (2021). 6-month neurological and psychiatric outcomes in 236 379 survivors of COVID-19: A retrospective cohort study using electronic health records. ***The Lancet. Psychiatry, 8***(5), 416–427. | **Information on the incidence of dementia among older adult controls was not available** |
| 14. | Brown, E. E., Kumar, S., Rajji, T. K., Pollock, B. G., & Mulsant, B. H. (2020). Anticipating and mitigating the impact of the COVID-19 pandemic on Alzheimer's Disease and related dementias. ***The American Journal of Geriatric Psychiatry : Official Journal of the American Association for Geriatric Psychiatry, 28***(7), 712–721. | **Narrative Review** |
| 15. | Rahmati, M., Yon, D. K., Lee, S. W., Soysal, P., Koyanagi, A., Jacob, L., Li, Y., Park, J. M., Kim, Y. W., Shin, J. I., & Smith, L. (2023). New-onset neurodegenerative diseases as long-term sequelae of SARS-CoV-2 infection: A systematic review and meta-analysis. ***Journal of Medical Virology, 95***(7), e28909. | **Systematic review with Meta-analysis** |
| 16. | Hariyanto, T. I., Putri, C., Arisa, J., Situmeang, R. F. V., & Kurniawan, A. (2021). Dementia and outcomes from coronavirus disease 2019 (COVID-19) pneumonia: A systematic review and meta-analysis. ***Archives of Gerontology and Geriatrics, 93***, 104299. | **Systematic review with Meta-analysis** |
| 17. | Pyne, J. D., & Brickman, A. M. (2021). The impact of the COVID-19 pandemic on dementia risk: Potential pathways to cognitive decline. ***Neuro-degenerative Diseases, 21***(1-2), 1–23. | **Narrative Review** |
| 18. | Gao, S., Burney, H. N., Callahan, C. M., Purnell, C. E., & Hendrie, H. C. (2019). Incidence of dementia and Alzheimer Disease over time: A meta-analysis. ***Journal of the American Geriatrics Society, 67***(7), 1361–1369. | **Meta-analysis** |
| 19. | Gordon, M. N., Heneka, M. T., Le Page, L. M., Limberger, C., Morgan, D., Tenner, A. J., Terrando, N., Willette, A. A., & Willette, S. A. (2022). Impact of COVID-19 on the onset and progression of Alzheimer's Disease and related dementias: A roadmap for future research. ***Alzheimer's & Dementia : The Journal of the Alzheimer's Association, 18***(5), 1038–1046. | **Narrative Review** |
| 20. | Olivera, E., Sáez, A., Carniglia, L., Caruso, C., Lasaga, M., & Durand, D. (2023). Alzheimer's disease risk after COVID-19: A view from the perspective of the infectious hypothesis of neurodegeneration. ***Neural Regeneration Research, 18***(7), 1404–1410. | **Systematic review** |

# **Table S4(a).** Quality assessment of included studies via the Newcastle-Ottawa Scale (NOS) for cohort studies.

| **Included studies** | **Selection (4 items)** | | | | **Comparability (2 items)** | | **Outcome (3 items)** | | | **Total score** |
| --- | --- | --- | --- | --- | --- | --- | --- | --- | --- | --- |
| **Author & Year** | **(1).**  **Representativeness of the exposed cohort** | **(2). Selection of the non-exposed cohort** | **(3). Ascertainment of exposure** | **(4). Demonstration that outcome of interest was not present at start of study** | **(1). The study controls for age, sex and marital status** | **(2). Study controls for other factors** | **(1). Assessment of outcome** | **(2). Was follow-up long enough for outcomes to occur** | **(3). Adequacy of follow-up of cohorts** |  |
| Cohen et al. 2022 | ★ | ★ | ★ | ★ | ★ | ★ | ★ | - | ★ | 8 |
| Gollop et al. 2023 | ★ | ★ | ★ | ★ | ★ | ★ | ★ | ★ | ★ | 9 |
| Liu et al. 2022 | ★ | ★ | ★ | ★ | ★ | ★ | - | ★ | ★ | 8 |
| Park et al. 2021 | ★ | ★ | ★ | ★ | ★ | - | ★ | - | ★ | 7 |
| Qureshi et al. 2022 | ★ | ★ | ★ | ★ | ★ | - | ★ | ★ | ★ | 8 |
| Taquet et al. 2021 | ★ | ★ | ★ | ★ | ★ | ★ | ★ | - | ★ | 8 |
| Taquet et al. 2022 | ★ | ★ | ★ | ★ | ★ | ★ | ★ | ★ | ★ | 9 |
| Wang et al. 2022 | ★ | ★ | ★ | ★ | ★ | ★ | ★ | ★ | ★ | 9 |
| Xu et al. 2022 | ★ | ★ | ★ | ★ | ★ | ★ | ★ | ★ | ★ | 9 |
| Zarifkar et al. 2022 | ★ | ★ | ★ | ★ | - | - | ★ | ★ | ★ | 7 |

# **Table S4(b).** Quality assessment of an included study via the Newcastle-Ottawa Scale (NOS), adapted for cross-sectional studies.

| **Included study** | **Selection (4 items)** | | | | **Comparability (2 items)** | | **Outcome (2 items)** | | **Total score** |
| --- | --- | --- | --- | --- | --- | --- | --- | --- | --- |
| **Author & Year** | **(1). Representativeness of the sample** | **(2). Sample size** | **(3). Non-respondents** | **(4). Ascertainment of the exposure (risk factor)** | **(1). The study controls for the most important factor** | **(2). The study control for any additional factor** | **(1). Assessment of the outcome** | **(2). Was follow-up long enough for outcomes to occur** |  |
| Liu et al. 2021 | ★ | ★ | ★ | ★ | ★ | ★ | ★ | - | 7 |

# **Figure S1.** Forest plot of the meta-analysis of the risk of new-onset cognitive impairments (including both CIND and dementia) between COVID-infected group and non-COVID-infected group.


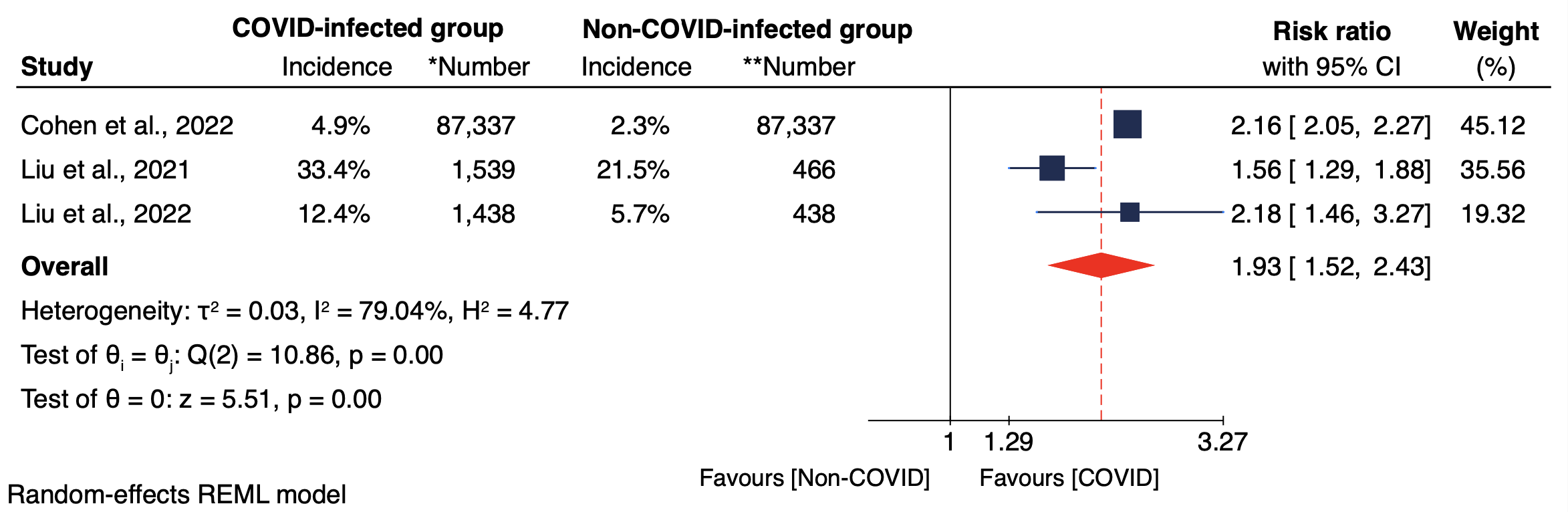


Abbreviation: CIND, Cognitive Impairment No Dementia

**Incidence** denotes the NOD rate within a specified group.

***Number** denotes the total number of participants in the COVID-infected group.

****Number** denotes the total number of participants in the non-COVID-infected group.

# **Figure S2.** Forest plot of the subgroup meta-analyses of the new-onset dementia risk in COVID-positive group, other respiratory infection group, and COVID-negative otherwise unspecified group, separately, based on sex (i.e., male vs. female).

**Events** denote the occurrence of NOD within a specified group.

# **Figure S3.** Forest plot of the meta-analysis of the new-onset dementia risk between non-COVID-hospitalized (i.e., outpatient) and COVID-negative groups.


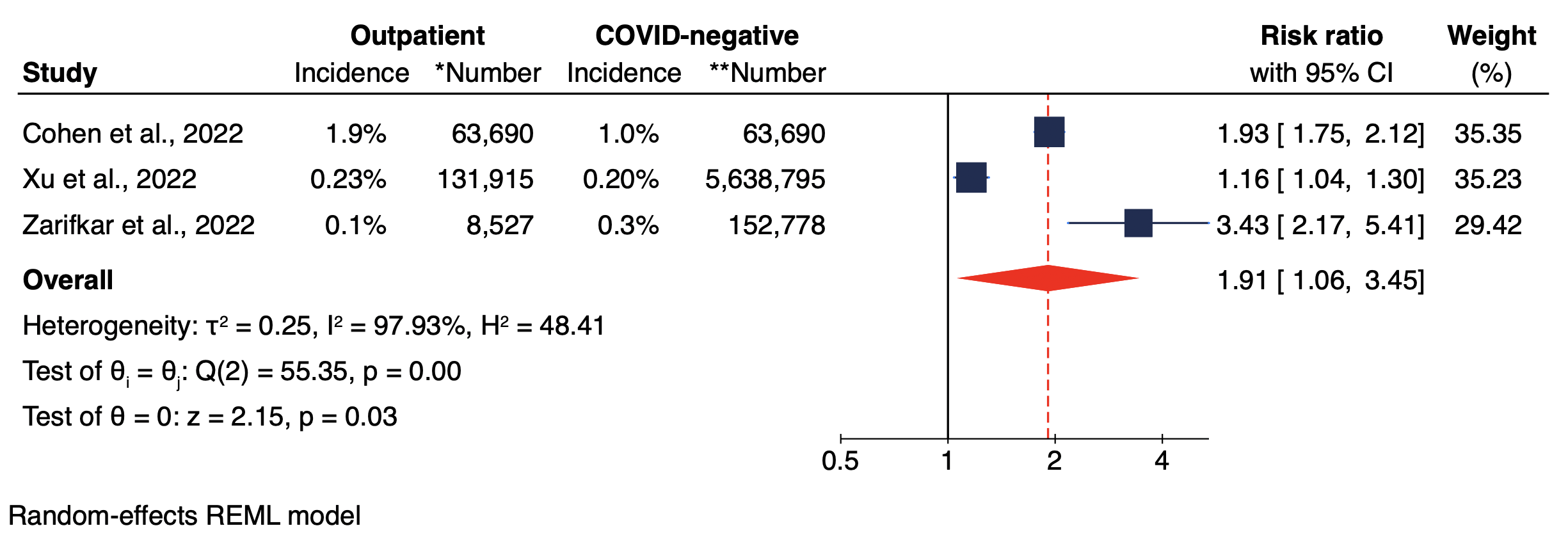


**Incidence** denotes the NOD rate within a specified group.

***Number** denotes the total number of participants in the non-COVID-hospitalized group.

****Number** denotes the total number of participants in the COVID-negative group.

# **Figure S4.** Forest plot of the meta-analysis of the new-onset dementia risk between COVID-hospitalized (i.e., inpatient) and COVID-negative groups.


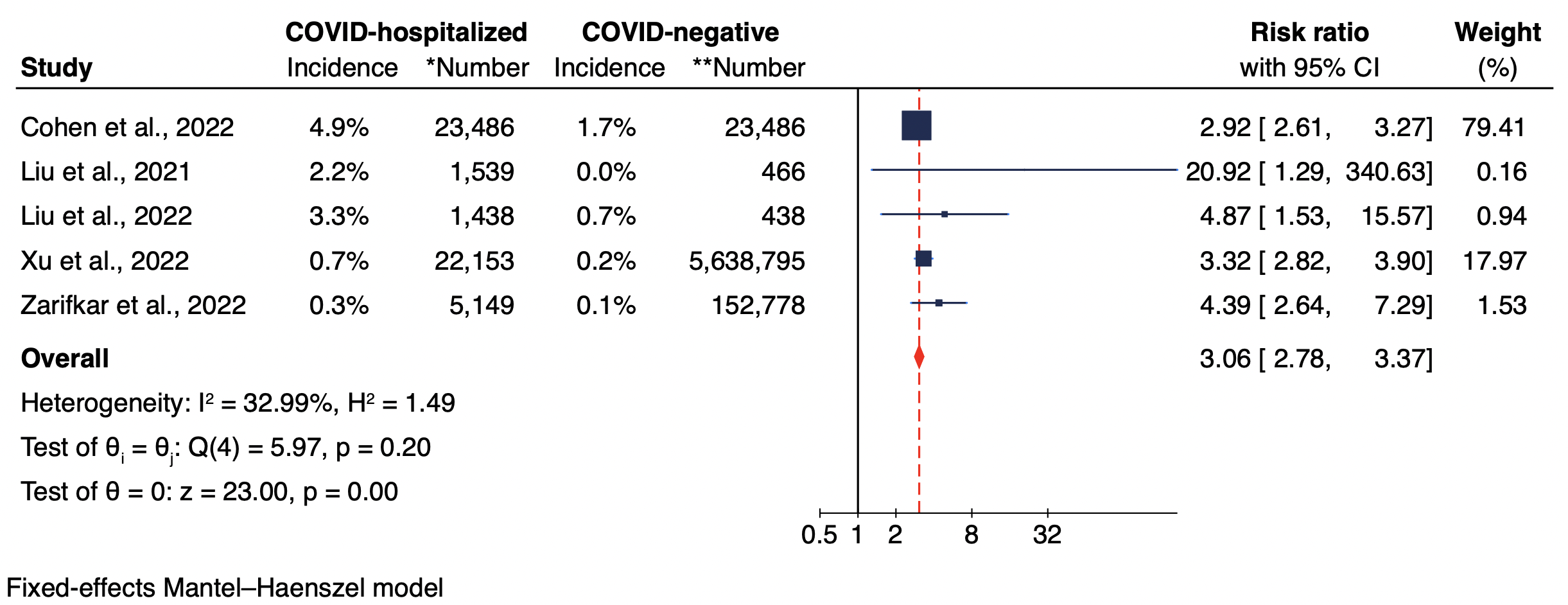


**Incidence** denotes the NOD rate within a specified group.

***Number** denotes the total number of participants in the COVID-hospitalized group.

****Number** denotes the total number of participants in the COVID-negative group.

# **Figure S5.** Forest plot of the meta-analysis of the new-onset dementia risk between COVID-infected group and non-COVID-infected group, across five studies employing Propensity Score Matching (PSM).


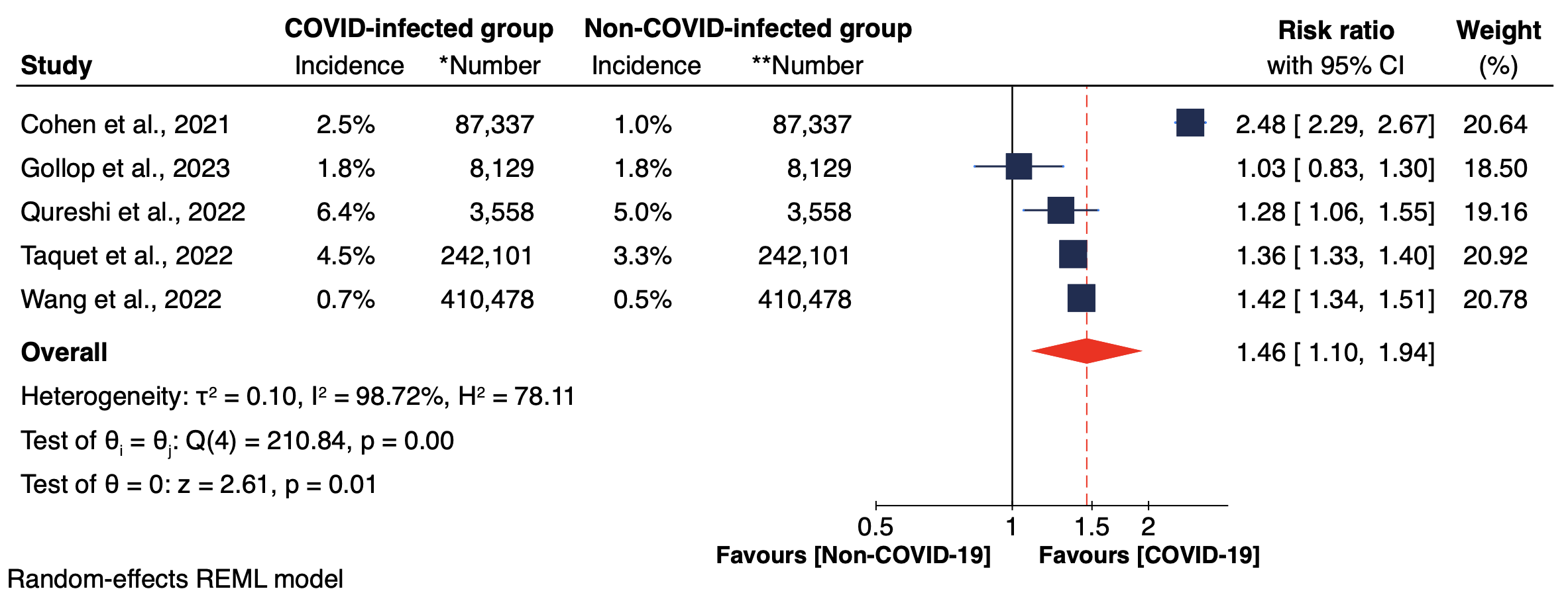


**Incidence** denotes the NOD rate within a specified group.

***Number** denotes the total number of participants in the COVID-infected group.

****Number** denotes the total number of participants in the non-COVID-infected group.

# **Figure S6.** L'Abbé plot.

# **Figure S7.** Galbraith plot.

# **Figure S8.** Random-effects meta-regression model.


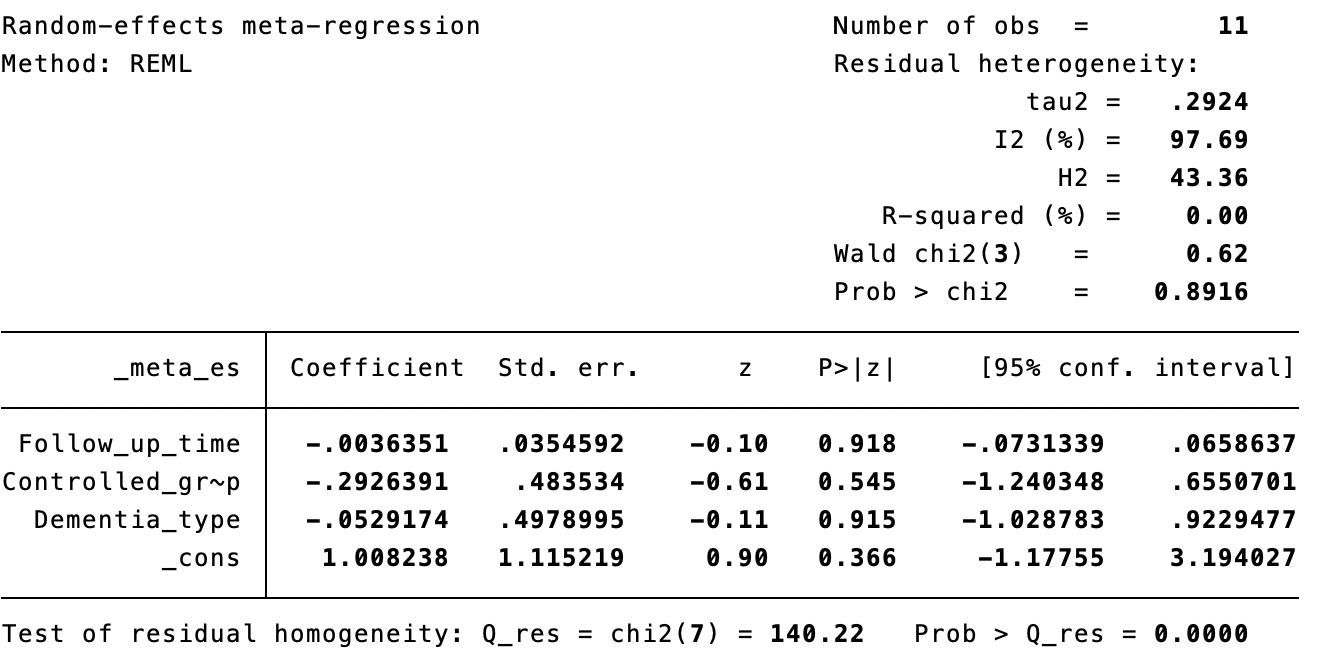


# **Figure S9.** Sensitivity analysis.

Abbreviation: m, months.

# **Figure S10.** Contour-enhanced funnel plot.
